# Gendered movement ecology and landscape use in Hadza hunter-gatherers

**DOI:** 10.1038/s41562-020-01002-7

**Published:** 2021-01-04

**Authors:** Brian M. Wood, Jacob A. Harris, David A. Raichlen, Herman Pontzer, Katherine Sayre, Amelia Sancilio, Colette Berbesque, Alyssa N. Crittenden, Audax Mabulla, Richard McElreath, Elizabeth Cashdan, James Holland Jones

**Affiliations:** 1 Department of Anthropology, University of California, Los Angeles, CA USA; 2 Department of Human Behavior, Ecology and Culture, Max Planck Institute for Evolutionary Anthropology, Leipzig, Germany; 3 Institute of Human Origins, School of Human Evolution and Social Change, Arizona State University, Tempe, AZ USA; 4 Human and Evolutionary Biology Section, Department of Biological Sciences, University of Southern California, Los Angeles, CA USA; 5 Department of Evolutionary Anthropology, Duke University, Durham, NC USA; 6 The Center for Health and The Social Sciences, University of Chicago, Chicago, IL USA; 7 Department of Life Sciences, University of Roehampton, London, UK; 8 Department of Anthropology, University of Nevada, Las Vegas, NV USA; 9 Department of Archaeology and Heritage, University of Dar es Salaam, Dar es Salaam, Tanzania; 10 Department of Anthropology, University of Utah, Salt Lake City, UT USA; 11 Department of Earth System Science, Stanford University, Palo Alto, CA USA

**Keywords:** Social anthropology, Biological anthropology

## Abstract

Understanding how gendered economic roles structure space use is critical to evolutionary models of foraging behaviour, social organization and cognition. Here, we examine hunter-gatherer spatial behaviour on a very large scale, using GPS devices worn by Hadza foragers to record 2,078 person-days of movement. Theory in movement ecology suggests that the density and mobility of targeted foods should predict spatial behaviour and that strong gender differences should arise in a hunter-gatherer context. As predicted, we find that men walked further per day, explored more land, followed more sinuous paths and were more likely to be alone. These data are consistent with the ecology of male- and female-targeted foods and suggest that male landscape use is more navigationally challenging in this hunter-gatherer context. Comparisons of Hadza space use with space use data available for non-human primates suggest that the sexual division of labour likely co-evolved with increased sex differences in spatial behaviour and landscape use.

## Abstract (web summary)

Wood et al. examine gender differences in Hadza hunter-gatherer spatial behaviour using 2,078 days of GPS-recorded travel. As predicted from principles of foraging ecology, Hadza men walked further per day, explored more land, followed more sinuous paths and were much more likely to be alone.

## Main

Our species’ use of space is exceptional in many regards. At a global level, our distribution across the earth’s habitats attests to an unrivalled capacity to explore and adapt to novel environments. Our spatial organization also reflects social institutions that organize our species’ exceptional levels of co-operation. One of these institutions is a sexual division of labour, which is observed in some form in all traditional human societies but seen in no other primate. Understanding the causes and consequences of gender-specialized work is an active area of research in evolutionary anthropology. Here, employing theory in movement ecology, we test predictions about the spatial consequences of the sexual division of labour using a very large corpus of GPS data collected among Hadza hunter-gatherers.

Sex differences in spatial behaviour are observed in many species, owing to sex-differentiated strategies of reproduction, territorial behaviour and foraging^1–3^. If persistent sex differences in spatial behaviour exist, corresponding sex differences in spatial cognition are also likely to have arisen through developmental and co-evolutionary processes^4,5^. Some basic measures of spatial cognition, including three-dimensional mental rotation and dead reckoning, have been studied in many human populations, and research to date shows that in these two measures, average male scores are characteristically higher than those of women^6,7^. Some have ascribed this result to our species’ evolutionary history of hunting and gathering^8^, while others propose that ranging patterns established prior to the advent of the sexual division could account for these sex differences^9^.

Recent cross-cultural studies of spatial behaviour and cognition have shown that gender differences reflect local environmental and cultural contexts, demonstrating the developmental plasticity and behavioural flexibility of our species^10–13^. For progress in this field, joint studies of spatial cognition and spatial behaviour across diverse samples of human societies and non-human species are called for. To date, most large-scale research into human mobility has been done in urban contexts, using low-resolution methods and often oriented towards statistical description rather than explanation^14,15^. Few studies currently exist quantifying people’s movement patterns in rural areas using GPS devices, although such studies are becoming increasingly common^16–20^. Compelling features of the current study are that we use ecological theory to make predictions about spatial behaviour and test these predictions using the largest corpus of GPS measurements available for any traditional subsistence society.

Among the Hadza of the Lake Eyasi region of northern Tanzania, men’s work involves hunting large and small animals and harvesting wild honey, and women’s work focuses on gathering plant foods. Classic ecological theory is a useful guide to the spatial challenges these jobs entail^21,22^. Higher trophic-level species, which Hadza men target, are generally expected to be less abundant than the plants (for example, tubers, fruit and greens) that women harvest. The actual availability of male- and female-targeted foods depends on the specific species sought and can vary considerably in space and time. Prior research with the Hadza indicates that the animals targeted by men are much less abundant and harvestable than female-targeted foods. Men hunt a variety of species, but ungulates such as dik dik (*Madoqua kirkii*) and impala (*Aepyceros melampus*) comprise a large portion of their hunting incomes^23,24^. Given an annual rainfall of ~ 500 mm year^−1^ (ref. ^23^), ungulate biomass in the Hadza region is expected to be only 30–50 kg hectare^−1^ (ref. ^25^); recent mammal monitoring suggests wild ungulate biomass is in fact lower^26^. By contrast, Vincent^27^ reports that two dietary staples targeted by Hadza women, the tubers //ekwa (*Vigna frutescens*) and shumuko (*Vatovaea pseudolablab*), were found at levels averaging 5,200 kg hectare^−1^ and 63,000 kg hectare^−1^, respectively. These data illustrate a stark difference in the availability of male- and female-targeted foods.

Harestad and Bunnel’s^28^ model considers the relationship between resource density and day range and generates one of our core predictions:

$${\mathrm{Day}}\,{\mathrm{range}}\, \propto \frac{{{\mathrm{Metabolic}}\,{\mathrm{needs}}}}{{{\mathrm{Resource}}\,{\mathrm{density}}}}$$

This simple formula states that day range (distance travelled per day) should be proportional to the ratio of metabolic needs (energy harvested per day) to resource density (density of food energy in the landscape). Applications of this formula to comparative movement data show that carnivore day ranges typically exceed those of herbivores, as we would expect given the lower resource densities of animal prey^28,29^.

By analogy, this model suggests that Hadza men should generally have higher day ranges and rates of land exploration, because the animal foods they target are much rarer than female-targeted foods. We also reasoned that men would be more likely to travel alone, for the sake of maintaining stealth while hunting. Hadza men’s hunting entails searching for and pursuing mobile resources, while women’s gathering entails harvesting immobile plant resources. Because Hadza men pursue mobile prey, we also expected their travel to be more sinuous than women’s, who would be more likely to follow direct trajectories to and from plant patches. Sinuosity (also called tortuosity) is defined as the ratio of an actual distance travelled between locations to the distance of the shortest as-the-crow-flies path. These expectations are based in theory but are all the more plausible given our close observations of Hadza while foraging^30–33^. Our large GPS database permits a more robust and objective test of these predictions than would a traditional anthropological study without the use of sensors.

The current study builds upon two prior studies of Hadza spatial cognition and behaviour. Cashdan et al.^3^ show that Hadza men scored higher than women in tests of navigational dead reckoning. That study did not include measures of spatial behaviour or navigational challenges experienced. In a prior study of Hadza movement patterns, Raichlen et al.^20^ investigated the distribution of step lengths (bouts of travel involving few turns or stops) within a much smaller sample of GPS tracks from four camps. That study found that men’s step length distributions were less likely than women’s to be best fit by a power law or truncated power law distribution (37% versus 57%). This result could indicate that women travelled more often in a quasi-straight line fashion, but that possibility was not examined.

Beyond foraging ecology, additional factors are expected to influence the travel patterns of Hadza men and women differently. During our field research, Hadza women routinely stated a desire to avoid dangerous animals or unfamiliar men when foraging (personal observations and Hawkes et al.^34^). Interactions between the Hadza and neighbouring Datoga pastoralists are historically hostile^35^ and can be intermittently tense today, and women often request the accompaniment of an armed man or older boy while foraging. This concern for safety is a further reason to expect that relative to men, women might travel in larger groups, in greater proximity to one another and closer to home. Extending this idea, we were interested in learning whether women would be more likely to be in close proximity to campmates when travelling far from home. We test this idea using measures of interindividual proximity. Women’s travel patterns were also explored to see whether the presence of young children (aged 2 years or younger) decreased their mothers’ daily travel, a possibility suggested by studies of other forager societies^36,37^.

The first goal of this study is to test whether, and to what extent, gender differences in Hadza space use arise as predicted by theory in movement ecology. To examine linkages between space use and spatial cognition, we calculate proxy measures of the daily navigational challenges confronted by men and women. These measures include distances travelled, rates of exploring habitat, the sinuosity of travelled routes and proximities between individuals while travelling. All else being equal, we assume that walking further distances, exploring more habitat, travelling on more circuitous routes and travelling alone are more navigationally challenging than travelling short distances, visiting few places, walking on direct routes or travelling in groups, where collective discussions aid navigation. We posit that spatial reorientations, in which a traveller takes note of their current trajectory of travel in relation to their prior route or their planned destination, could be calculations that draw on similar cognitive processes as those measured in tests of three-dimensional mental rotation or perspective taking. Consistent with this idea, laboratory measures of spatial ability, including mental rotation, are positively correlated with subjects’ ability to navigate and learn real and virtual environments^38,39^.

Lightweight GPS devices provide an excellent opportunity to assess spatial behaviour across cultural and ecological contexts. In Methods, we detail how we collected, stored, processed and analysed the Hadza spatial database. Each statistical model in the main text is numbered, and the details of each model are listed in the Supplementary methods.

## Results

### Distances and steps per day

Here, we compare distances and steps walked per day over 1,097 days of female travel and 981 days of male travel. These data are derived from 179 Hadza research participants (women and girls accounted for 87 of the participants and men and boys, 92) ranging in age from 2 to 84 years (mean = 36, s.d. = 19).

**Fig. 1:**
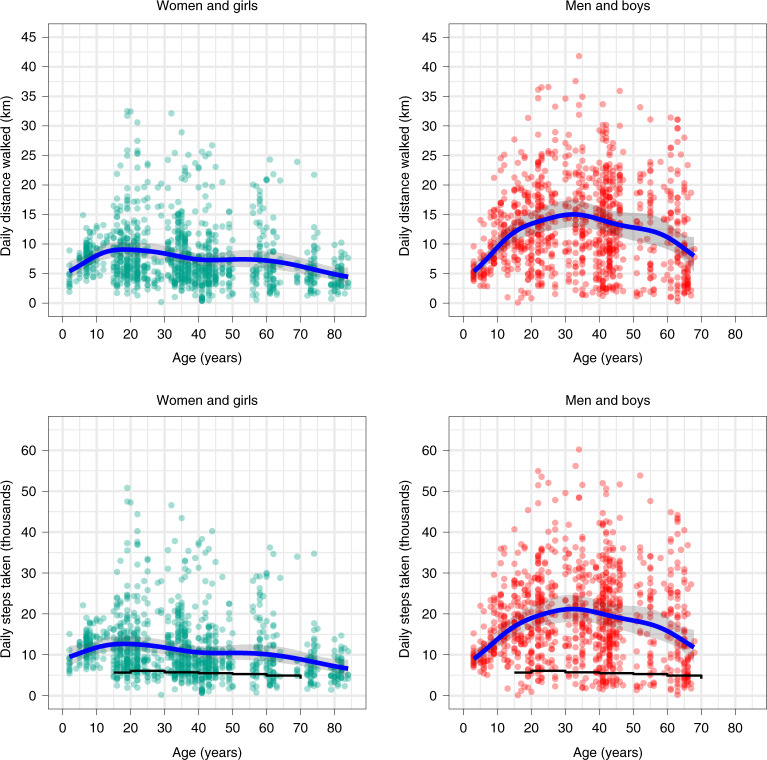
Hadza daily distance walked and steps taken by gender and age. Daily distance walked (top) and steps taken (bottom) by gender and age. The blue trend lines in the upper and lower panels represent the expected Hadza means and their 95% credible intervals (shaded areas) from models 1 and 2, respectively. In the bottom panels, the black lines are the average step counts of 717,527 smartphone users in 111 nations^45^. The data measure the daily travel of 92 men and boys (combined) and 87 women and girls (combined), representing 1,097 days of female travel and 981 days of male travel (Table 1).

**Extended Data Fig. 1:**
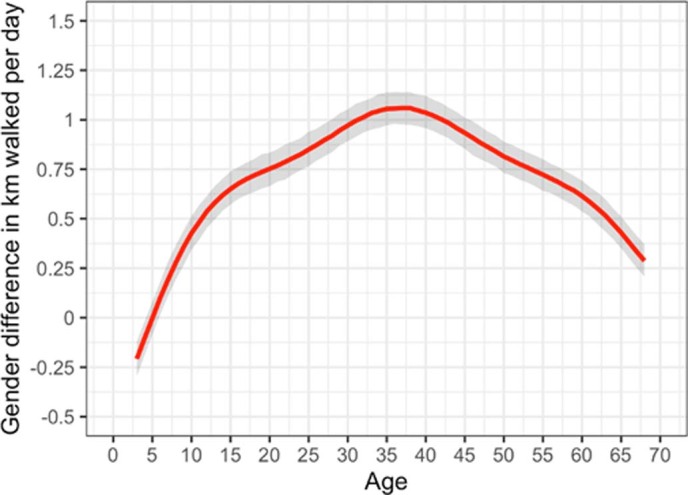
Gender difference in distance walked per day by age. Standardized (Cohen’s D) gender difference in distance walked per day by age. The means and standard deviations used to calculate Cohen’s D are derived from simulations from the posterior distribution of model 1. The red line represents the mean estimate of D for each age, and the grey ribbon represent the 95% quantile credible interval of the mean. See method section “Distance walked” for more info. Model 1 was fit to the GPS data of 92 males and 87 females, representing 1,097 days of female travel and 981 days of male travel (see Table 1).

In examining Fig. 1, distinct age trends in Hadza daily mobility appear for each gender. Among boys, a steep trend of increasing travel extends from the earliest ages to around 15 years of age. This increase is steeper among boys than girls, and thus a gender difference in travel emerges early. At 6 years of age, boys walked greater distances per day than girls on average (Cohen’s *d* = 0.10, 95% credible interval (CI) = 0.02–0.18; Extended Data Fig. 1 and Supplementary Table 5), and by 10 years of age, this difference was more pronounced (Cohen’s *d* = 0.43, 95% CI = 0.34–0.51). At 15 years of age, boys walked 12 km per day on average and girls, 9 km (Cohen’s *d* = 0.65, 95% CI = 0.57–0.74; Supplementary Table 5). After its early emergence, a gender difference in travel persists across the life course. The gender difference is highest in ages 30–45 years, when model 1 estimates that on average, men walked ~14 km per day and women walked ~ 8 km per day.

The lifetime pattern of daily travel in Hadza men forms an inverted U pattern, with maximum travel observed near age 32 years (~15 km day^−1^). For women, a more complex pattern is apparent. An early peak is seen around ages 17–20 years (~9 km day^−1^), followed by a decline until 40 years of age and then a plateau through age 60 years (~7 km day^−1^). The sustained physical activity of postmenopausal Hadza women highlighted by prior work is reflected in our GPS data^34,40^. Between ages 60 and 68 years, a steeper decline in mean distance walked per day is seen among men than women: the decline is 3.2 km (95% CI = 2.57–3.85) among men and 0.72 km (95% CI = 0.35–1.05) among women. This causes an attenuation of the gender difference, which at age 68 years is Cohen’s *d* = 0.29 (95% CI = 0.21–0.37; Supplementary Table 5 and Extended Data Fig. 1).

The decline in women’s travel after their late teens seen in Fig. 1 could be owing to the demands of child care, as the average age at first birth for Hadza women is 19 years, and subsequent years are associated with growing families and increasing levels of child dependency^41^. Other studies^42^ have found that young children restrict women’s travel. To test for this possibility, we examined the daily travel of women with and without young children (aged 2 years or younger) under their care. The difference in means at each age was calculated from the posterior distribution of model 3, with individual and camp-level random effects held at their average values.

**Fig. 2:**
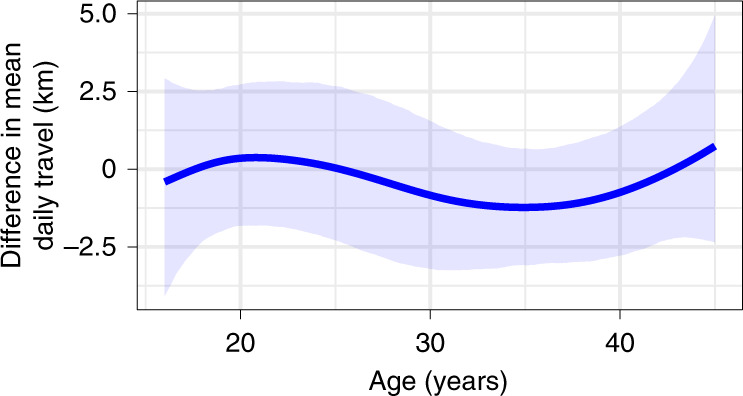
Difference in the mean daily travel of women with and without children aged 2 years or younger under their care, calculated from model 3. The blue line represents the expected difference in mean daily travel (that is, mean with children minus mean without children), and the shaded area is its 95% CI. Model 3 was fit to GPS data from 19 women aged 16–45 years with young co-resident children and 39 without (Supplementary Table 3).

As seen in Fig. 2, our analysis does not reveal a strong impact of young child dependency on women’s travel. The 95% CI of the difference of means between these two groups overlaps zero across all ages; at age 30 years, for example, the difference in means is estimated to be −0.84 km (95% CI ranging between −3.21 and 1.55). There is a suggestion of a possible decline in travel for women aged between 30 and 40 years that bears further examination (Discussion). The model that includes a term for child dependency has lower out-of-sample predictive accuracy, as measured by the widely applicable Bayesian information criteria (WAIC^43^). The WAIC value of model 3 is 3,828.2, indicating lower predictive accuracy than a simpler null model that does not include a term for child dependency (model 3 reduced, WAIC 3,823.4).

Using GPS-based estimates of step counts^44^, our data suggest that across the ages of 18–75, Hadza men and women walked on average 18,434 and 10,921 steps per day, respectively. In a global sample of smartphone users in middle- and high-income countries^45^, men’s step counts in these ages averaged 5,332, which is less than a third of Hadza men’s totals, and women in this sample logged 4,064 steps on average, less than half Hadza women’s average.

### Land explored

**Fig. 3:**
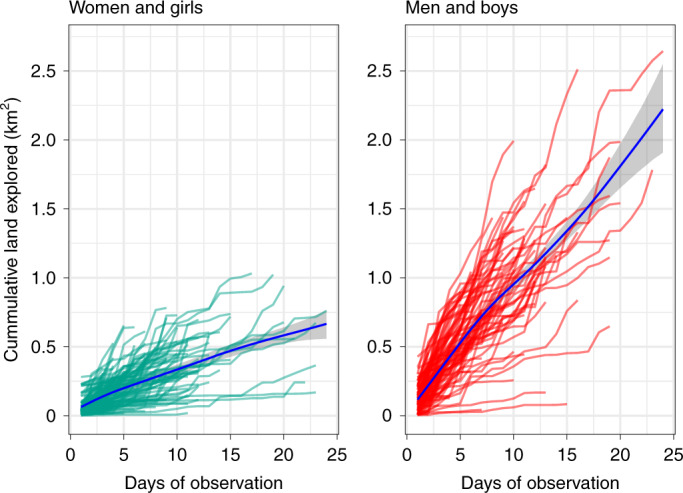
Cumulative land explored by Hadza individuals across days of observation. Each line represents one person in one camp. The blue trend line is the estimated mean, and the shaded area is its 95% CI from fit model 4. The data measure the land explored by 92 men and boys and 87 women and girls, representing 1,097 days of female travel and 981 days of male travel (Table 1). See “Land exploration” in Methods for more details of this analysis.

We predicted that men, seeking rarer and more mobile resources, would be more exploratory in their travel than women, who seek more abundant, immobile resources. One way to be more exploratory is to walk greater distances (Fig. 1). Another way is to distribute one’s travel more expansively. More expansive travel would result in a person visiting more parts of the landscape per day and per meter travelled. To test this idea, we conducted raster-based analyses (see “Land exploration” in Methods). In Fig. 3 we display the area of land explored by each person in each camp for each day of observation.

Figure 3 shows a clear gender difference in the expansiveness of travel, as predicted. On average, women and girls visited about the same area of landscape after 15 days (mean = 470,720 m^2^, 95% CI = 427,535–515,050) that men and boys visited after only 5 days (mean = 528,478, 95% CI = 493,007–564,014). This pattern is consistent with our predictions based on the lower environmental density and higher mobility of male-targeted foods^23,26,27,46^. Table 1 summarizes raster measures of land utilization in each camp. To measure how distinct male and female landscape use were, we calculated geographic segregation by gender. This measure is based on the extent of overlap between male-visited and female-visited parts of the landscape.

**Table 1:** Raster measures of total area of unique land (km^2^) visited by gender in each camp and the extent of spatial overlap between male- and female-visited areas (geographic segregation)

Camp	Year	Total land visited^a^ (km^2^)	Male-visited land (km^2^)	Female-visited land (km^2^)	Male: female ratio	Land visited by both genders studied (km^2^) (% of total land visited)
Setako	2009	552.7	462.1	156.9	2.9	66.2 (12.0%)
Setako	2010	281.4	223.3	97.3	2.3	39.2 (13.9%)
Sengeli	2010	510.3	485.7	57.9	8.4	33.4 (6.5%)
Sanola	2014	656.7	594.1	172.9	3.4	110.4 (16.8%)
Sengeli	2015	548.4	453.9	137.2	3.3	42.7 (7.8%)
Buruku	2015	434.4	371.2	121.8	3.0	58.6 (13.5%)
Buruku	2016	622.7	585.0	96.8	6.0	59.1 (9.5%)
Hukumako	2016	1,200.7	1,109.7	319.6	3.5	228.7 (19.0%)
Kideru Juu	2016	539.0	480.8	124.1	3.9	65.9 (12.2%)
Hukumako	2017	344.3	282.4	131.7	2.1	69.8 (20.3%)
Ol Piro	2018	885.9	845.9	125.8	6.7	85.8 (9.7%)
Hukumako	2018	360.1	287.2	157.4	1.8	84.5 (23.5%)
Mean		578.0	515.1	141.6	3.9	78.7 (13.6%)

^a^In this analysis, land visitation is measured by summing the 10 m^2^ raster cells intersected by GPS tracks.

**Fig. 4:**
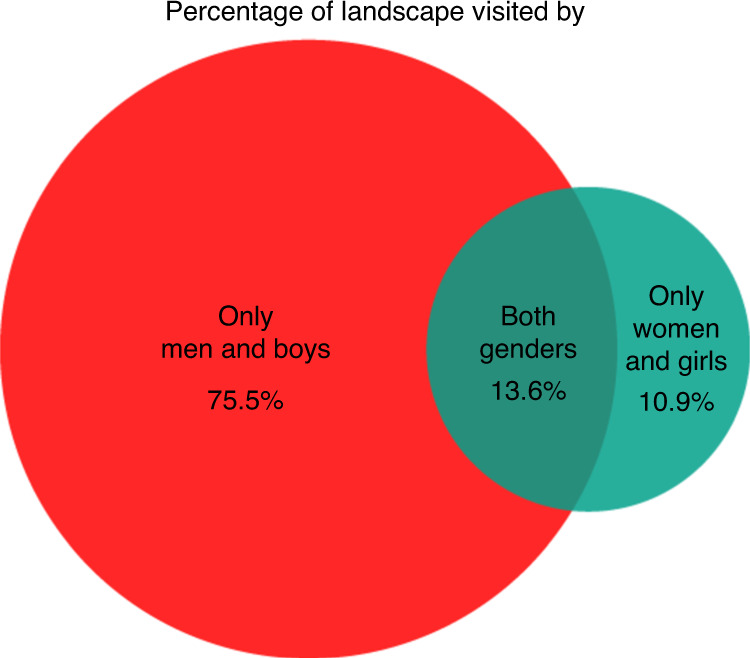
Geographic segregation by gender. The landscape area visited by only men, by only women or by both genders was calculated for each camp (*n* = 12). The average value across camps, expressed as a percentage of the total land area visited, is plotted here. See Table 1 for camp-level values and “Geographic segregation by gender” in Methods for more analysis details.

Table 1 shows that in every camp observed, men and boys visited more of the landscape than women and girls (Wilcoxon’s signed rank test, *z* = 78, *P* < 0.001, 95% confidence interval of 227.95–519.75; *n* = 12). When averaged across camps, the area of land visited by men and boys was 3.9 times larger than the female-visited land area. These analyses also show that men and women occupy very distinct parts of the landscape. On average, only 13.6% of the total land visited was visited by both genders studied (that is, by at least one man and one woman); the average spatial segregation by gender is displayed in Fig. 4.

**Fig. 5:**
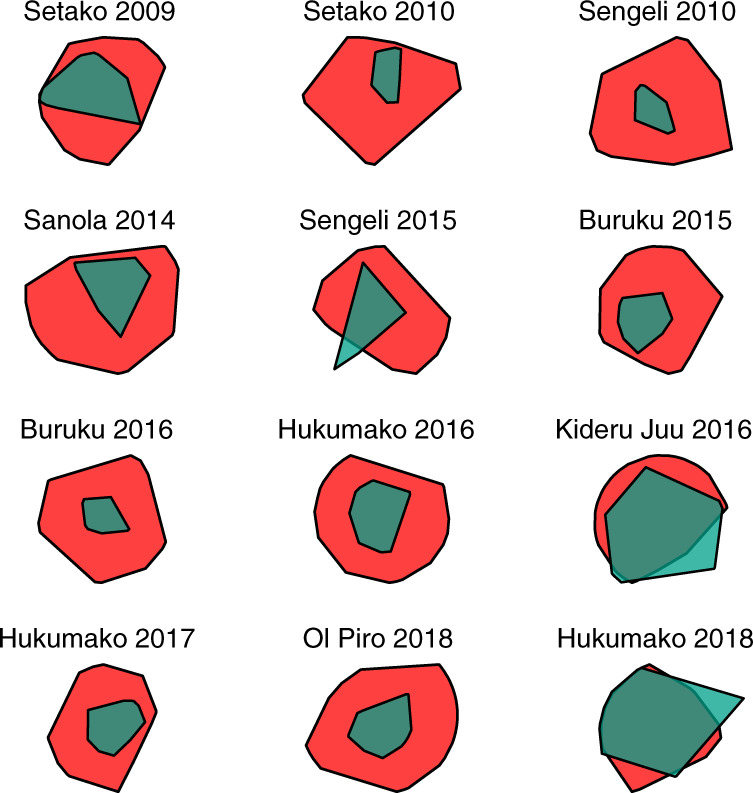
Male and female landscape use measured by MCPs across all studied camps. The MCPs for the 12 camps studied are shown, labelled with camp name and year of observation. Male landscape use is shown in red and female landscape use in green. The scale of each plot is constant within each camp but not across camps. See “MCP areas” in Methods for more details and Supplementary Table 6 for quantitative measures across camps.

Another common way to characterize space use is to calculate minimum convex polygons (MCPs) that surround sets of GPS locations. Figure 5 displays MCPs encompassing the track points of all male and female tracks in each camp.

The MCPs shown in Fig. 5 attest to men exploring more land than women. Across camps, the average ratio of male to female MCP area is 4:1, which is very similar to the ratio of 3.9:1 found in the more detailed raster-based measures. It is clear that male travel was more extensively distributed across landscapes. Extended Data Fig. 4 plots one week of travel each for the man and woman whose travel most closely matches the mean land exploration rate of their gender, and Supplementary Fig. 3 displays an overview of male and female tracks from one camp.

### Sinuosity of travel

**Fig. 6:**
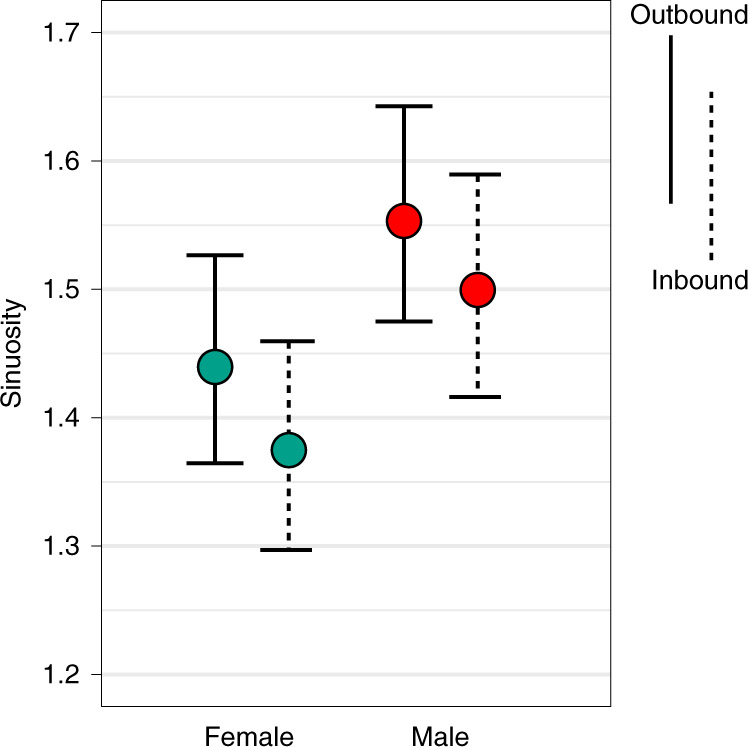
Outbound and inbound sinuosity by gender calculated using samples from the posterior distributions of models 5 and 6. Filled circles show mean sinuosity and error bars show 95% CI. Mean model-estimated outbound sinuosity (model 5) was 1.55 (95% CI 1.47–1.64) for men and boys and 1.44 (95% CI 1.36–1.53) for women and girls, and during inbound segments (model 6) it was 1.50 (95% CI 1.42–1.59) for men and boys and 1.38 (95% CI 1.30–1.46) for women and girls. Models 5 and 6 were fit to all days of travel in which research participants walked at least 500 m from camp, resulting in a sample of 1,526 tracks (763 male tracks, 763 female tracks).

Sinuosity was calculated to assess travellers’ need to reorient themselves while foraging and compute relationships between their current route, their prior route and their intended destination (see “Sinuosity of travelled routes” in Methods for details). In sinuous travel, such reorientations are expected to be more frequent. Both the raw data and the multilevel statistical models show that male travel was more sinuous than female travel, both when walking away from camp (outbound travel segments) and when walking towards home (inbound segments). In the raw data, male outbound sinuosity averaged 1.60 while female outbound sinuosity averaged 1.49 (Wilcoxon signed-rank test, *W* = 243,710, *P* < 0.001, 95% CI ranged from −0.112 to −0.051). During inbound segments, male average sinuosity was 1.57 and female inbound sinuosity, 1.45 (*W* = 230,616.5, *P* < 0.001, 95% CI ranged from −0.117 to −0.065). Estimates of mean sinuosity from our hierarchical statistical models 5 and 6, which include individual and camp-level random effects, are shown in Fig. 6.

**Extended Data Fig. 2:**
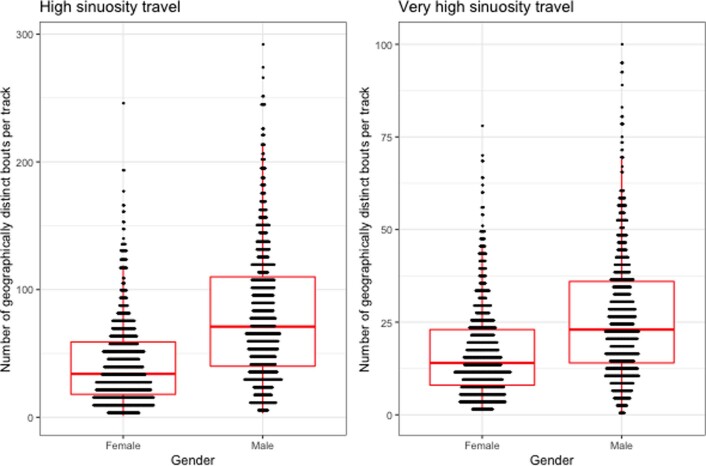
Gender difference in the sinuosity of travel. Counts of places where male and female travel was characterized by greater than average sinuosity (**a**) and higher than 99% quantile sinuosity (**b**). The distribution of these counts for all tracks is plotted, grouped by gender. The black dotplots show the distribution of binned raw scores, while the red boxplots summarize these distributions. The boxplots display the 25th, 50th (median) and 75th percentiles of the distributions. The upper whiskers extend from the 75th percentile to the largest value no further than 1.5 * IQR from the 75th percentile (where IQR is the inter-quartile range). The lower whiskers extend from the 25th percentile to the smallest value at most 1.5 * IQR from the 25th percentile.

The routes taken by both men and women were more direct when travelling back to camp (Fig. 6). This finding accords with our observations while carrying out focal follows of foraging Hadza; travel back to camp is often more determined and involves less searching for resources. This gender difference in behaviour revealed by our analysis of sinuosity is predicted from the foraging model in which men’s targeting of rarer and more mobile resources requires more exploration^28,29^ and the use of more circuitous paths during search and pursuit. Geographically weighted analysis also shows that men engaged in sinuous travel over more unique areas of their landscapes (Extended Data Fig. 2).

### Physical proximities while foraging

**Fig. 7:**
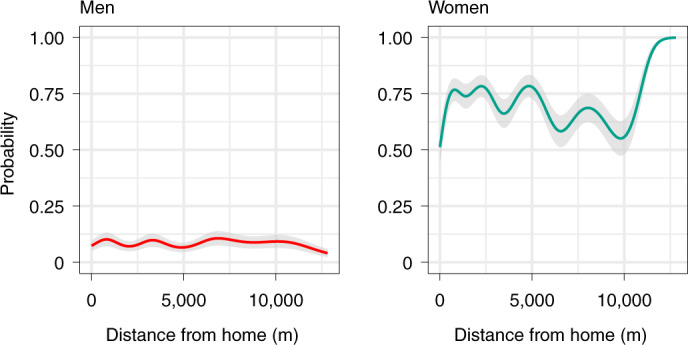
The probability of men and women being within 5 m of a campmate as a function of distance from home. The plot displays estimates from model 7. The lines and shaded areas represent the mean and ±1 s.e.m., respectively. The sociality sample includes 193,244 recordings of location and proximity by 178 individuals (85 male, 93 female) across 172 days of observation. The model estimates that when 2 km from camp, women had a 0.77 probability (s.e.m. = 0.05) of being near a campmate, and men had a much lower probability of 0.07 (s.e.m. = 0.02). When much farther from camp, at 12 km, these probabilities are 0.99 (s.e.m. = 0.01) for women and 0.06 (s.e.m. = 0.02) for men.

The sociality sample is a stratified subsample of our total Hadza spatial database. It consists of 197,244 samples of the physical proximities of all camp members wearing GPS devices. Across the entire sociality sample, we calculated whether target individuals were within 5 m of their nearest neighbour. To test the hypothesis that women are more social than men while foraging, we calculated summary statistics from the sample and carried out hierarchical modelling. In the sociality sample, the median distance observed between women and girls and their nearest neighbour (of either gender) was 3.2 m, while for men and boys, the median distance was 63.3 m (Supplementary Table 4). Using our hierarchical model (model 7), we statistically controlled for the influences of age and the number of people wearing GPS devices by holding them all at their mean values. From this model, we plot in Fig. 7 the probability of men and women being within 5 m of a campmate as a function of their distance from camp.

The estimates from model 7 shown in Fig. 7 demonstrate much closer proximity of foraging women, and thus, greater opportunities for women to make collective navigation decisions. The model estimates also show that women were more likely to be in close proximity to a campmate when they were far from camp (defined as beyond 10 km). These results generally accord with our expectations based on considerations of both foraging and personal safety.

## Discussion

Our study demonstrates that substantial gender differences in spatial behaviour were present across the life course in this hunter-gatherer society. Among the Hadza, men travelled further, visited more areas of the landscape, followed more sinuous routes and were more solitary while foraging. These patterns are consistent with the gendered ecology of hunting and gathering, in which men search for rarer, more energy-dense and more mobile resources than women^23,26,27,46^. These metrics of spatial behaviour also suggest that male-typical travel is more navigationally challenging. An earlier study^3^ showed that Hadza men scored higher on average than Hadza women in measures of spatial cognition and performance, a result that is consistent with the gender difference in spatial behaviour seen here. Gender differences in Hadza spatial behaviour emerge early; by the age of 6 years, boys are travelling slightly further on a daily basis (Cohen’s *d* = 0.1, 95% CI = 0.02–0.18; Supplementary Table 5). This means that the developmental environments that boys and girls experience are spatially distinct from an early age, and they continue to be so across the life course. It is important to note the following limitation of this study: as with most studies of gender differences, there is no way for our study to disentangle whether gender differences in spatial cognition or performance emerge solely from differences in developmental environments or are also impacted by innate physiological differences. Future work that examines changes in Hadza spatial behaviour and cognition in those areas where the traditional hunting and gathering economy has declined may provide useful insights into the consequences of different developmental environments.

The emergence of a gender difference in Hadza spatial behaviour around 6 years of age is broadly similar in timing to meta-analysis results^7^ showing that gender differences in spatial cognition emerge in the years 7–14 in Western samples. A study of children in the UK reported that gender differences in both exploratory behaviour and spatial cognition are detectable in the age range of 6 to 11 years^47^.

The Hadza’s highly mobile lifestyle is readily apparent in our data. Adult Hadza women walked an average of 7.6 km per day and logged 10,888 steps, while men walked 12.9 km and logged 18,476 steps. For comparison, a GPS study in Pretoria, South Africa^48^ found that urban women and men walked much less (2.9 and 3.9 km per day, respectively). Mbendjele women in the Republic of Congo, who practise a mixed farming and foraging lifestyle, also appear to have walked less than Hadza women (median of 4.35 km per day), but the Mbendjele study^18^ differed from ours in recording only travel out of camp, for 5.2 hours per day on average, while our study records all travel, for an average of 11.49 hours per day. Hadza women’s step counts were about twice as high as average smartphone-using subjects enrolled in a 111-nation study^45^, and Hadza men’s about three times higher than men in this global sample from middle- and high-income countries. Other rural, non-industrial societies may walk even more than the Hadza: a study of an Old Order Amish^49^ community in Ontario, Canada reported that adult women and men logged 14,196 and 18,425 steps per day, respectively.

It is interesting that we did not find clear statistical evidence that young children limited how far their mothers walked each day. Perhaps because Hadza plant foods are so abundant, there was little foraging incentive for a woman without young children to travel faster or further than another woman in her party carrying a small child. It seems equally plausible that women with small children are incentivized to keep up with the group. A recent study among the Twe of Namibia also found no difference in daily distances travelled between nursing and non-nursing reproductively aged women^19^. A limitation of this study is that subadults were not sampled as frequently as adults, for reasons discussed in Methods. In a future study, we hope to examine more fully the coupled patterns of movement by women and their children.

A limitation of our study is that we identified two features of socioecology that are likely to limit Hadza women’s travel relative to men’s: the fact that plant foods are immobile and found in much greater abundance than men’s foods, and the fact that women appear more fearful of encountering Datoga pastoralists while foraging out of camp. Our sense, based on many years of ethnographic research, is that the Datoga threat is kept in check by armed Hadza men and older boys who accompany parties of foraging women, and that the gender differences in spatial behaviour we identify here are by and large owing to the foraging ecology. A future study that examines movement patterns in areas with more or less Datoga presence should shed light on this issue.

The strong gender differences in space use observed here have implications for disease ecology and health measures. Our data show that men travelled more expansively (Fig. 3, Table 1 and Fig. 5), so one could reasonably assume that they were exposed to more diverse pathogens. Men are also more likely to be exposed to zoonotic pathogens because of interacting with and butchering animals. Sex differences in host immune responses are reported for many species, including humans^50^. Spatial data like those presented here should factor into causal modelling of such differences.

As evolutionary anthropologists, we are compelled to ask how these data can inform reconstructions of the past. Patterns of behaviour observed in diverse samples of contemporary hunter-gatherers are more likely to have been a part of our species’ evolutionary history than are patterns of daily life observed among university students or citizens of rich nation-states, who today make up most research subjects in social science^51^. The broad gender differences in spatial behaviour that we observed among the Hadza are likely to have been a regular feature of hunter-gatherer societies in the past and to also be present today where gendered foraging persists. Cross-cultural analyses show that hunting is a male-typical activity, and that when women do hunt, they tend to focus on smaller and less mobile prey^46,52,53^. The male hunting specialization is not a function of contemporary gender roles per se, as it is also consistent with signs of lower limb morphology and wear seen in prehistoric skeletal samples^54^. Interestingly, among non-human primates that occasionally hunt vertebrates, including chimpanzees, baboons and capuchins, males hunt more frequently than females^55–57^.

Over the last 2.5 million years, diets in the genus *Homo* shifted to include more hunting and pursuit of mobile foods^53,55,56,58^. These shifts undoubtedly increased home range sizes and are likely to have increased sex differences in spatial behaviour. Among living apes, sex differences in ranging are comparatively modest and reflect sex differences in reproductive ecology and territory defence. Male orangutans maintain larger home ranges than females despite similar diets, as males apparently seek increased access to females^59^. In chimpanzees, males travel an average of ~20% farther than females each day (3.6 versus 3.0 km per day), reflecting males’ larger foraging group sizes and territory defence^60^. Mountain gorilla males, whether solitary or in groups, have similar daily travel distances to females^61^. Studies of non-human primate ranging using GPS devices are rare, but GPS data from a recent study of olive baboons^62^ show little to no evidence for a sex difference. By contrast, the day ranges of Hazda men are 14.3 km per day at age 25 years and are 62% higher than those of Hazda women (Supplementary Table 3). An increase in gender-differentiated foraging over the last 2.5 million years may have increased sex differences in spatial cognition. More studies of spatial behaviour and cognition among non-human primates—especially apes—would be very helpful for constructing maximally plausible models of hominin cognitive evolution. At present, this interspecies analysis is limited to only a few studies in which precise spatial measures have been collected.

Our study has demonstrated a high degree of gender segregation in Hadza landscape use. It is worth remembering that this segregation out of camp is bookended with intense sociality and co-operation in camp among all co-residents^24^. Like other hunter-gatherer societies, the Hadza practise central place foraging, which anchors people’s movements to a camp and socially embeds them into a co-operative network of neighbours. Over evolutionary time, central place foraging and language would have fundamentally changed our genus’s spatial strategies. Among much else, it would have permitted early humans to form much vaster and dynamic mental models of landscapes by incorporating others’ spatial knowledge and their own experiences. It is routine for Hadza to exchange information at the end of the day, detailing their daily travels and experiences, and men and women often relay to one another any promising signs of plant or animal life they encounter. These derived features of human sociality mean that our spatial strategies are likely to differ strongly from those of other primates, above and beyond the role played by gender-differentiated foraging.

Research in movement ecology is examining space use with sophisticated methods and theory from diverse fields^18,62–66^, and these tools are increasingly being used to examine human behaviour. Such studies, carried out across diverse cultural contexts, will allow researchers to identify regular features of human spatial behaviour and shed light on spatial adaptations to varying climatic, economic and epidemiological conditions.

## Methods

### Research participants

During the fieldwork for this research, we sought out those Hadza camps where people were subsisting from hunting and gathering for the vast majority of their diet. We did not carry out work among camps where wage labour or tourism was occurring. During our research periods, we estimate that 90–100% of the calories consumed in these camps were derived from wild foods. The remaining fraction was comprised of agricultural products acquired through trade or food aid distributed by missionaries or government programmes. In Supplementary Table 1, we tabulate demographic information about research participants in this study.

Approval for this research was provided by all governing organizations (Institutional Review Boards at Harvard University, Yale University, Hunter College, the University of Arizona and University of California, Los Angeles as well as the Tanzania Commission for Science and Technology and the National Institute for Medical Research in Tanzania). All research participants provided their informed consent prior to participating in this project.

### The form and function of Hadza daily travel

In the camps under study, we lived with the Hadza and carried out focal follow observations of random samples of subjects, closely observing people throughout their day^24,30–32,40^. We also made daily records of foods that were brought back to each camp and carried out short interviews at the end of the day. All of these data collection activities and daily observations allow us to describe, in general terms, the kinds of activities that took place during the Hadza’s GPS-recorded travel. By far, the primary reason for travel out of camp was to search for and harvest food. The Hadza we lived with very seldom had food surpluses stored in their homes, and travelling out of camp to hunt and gather was a daily job that nearly all adults were engaged in. Travel out of camp was also routinely undertaken for collecting water and firewood. Much less frequently, travel was undertaken to visit neighbouring Hadza camps or for visiting more settled villages to carry out periodic trade and market activities. Though it is difficult to be precise about these things, our conservative estimate is that more than 95% of the forays out of camp that we recorded with GPS were driven by the goal of searching for and harvesting basic resources (food, water and firewood).

### GPS data collection and processing

The collection, storage, processing and analysis of large GPS datasets present many methodological challenges. To address these challenges, we have adopted techniques used in movement ecology research and developed custom measures and software suited for our research questions.

The total dataset includes 2,078 person-days (23,872 person-hours) of GPS-measured movement. This includes 1,097 person-days of female movement and 981 person-days of male movement. GPS data were collected by asking Hadza in 15 camps to wear small GPS devices (manufactured by Garmin, BadElf and Canmore). We continued GPS data collection for this study until we reached an arbitrary target of at least 2,000 total GPS tracks, a sample size substantially larger than acquired for any published study of human movement in a traditional society. During our data collection, we endeavoured to place GPS devices on a random sample of adult camp members each day. One or two researchers would walk through camp early in the morning as people were rousing. We would greet people at their homes or hearths and hand out GPS devices to be worn during the day. We varied the path we would walk through the camps so as to randomize which individuals would wear devices throughout the study. The devices were worn during the daylight hours, typically attached to belts or upper arms. Devices were likewise removed by researchers around nightfall, when most people had returned to camp and were retiring for the evening. In the analysis reported here, we only include data from subjects whose GPS devices recorded data for at least eight hours. The criterion of a minimum of eight hours allows for meaningful comparisons of spatial behaviour across the normally physically active hours of the day. In this sample of 2,078 tracks, the GPS devices recorded data for 11.49 hours on average (s.d. = 2.04). Data collection and analysis were not performed blind to the hypotheses of the study.

On average, devices were put on at 07:45:52 local time (East Africa Time, Coordinated Universal Time (UTC) + 3:00) and stopped recording at 18:57:53. On some occasions, individuals did not return to camp by early evening but stayed out of camp, usually to hunt at night or to visit a neighbouring Hadza settlement. In these cases, the GPS devices were not collected until their wearers returned (usually the next morning).

The total number of GPS devices used each day varied throughout the study, as a function of funding (how many we could purchase for a field season) and weather (how many devices we could charge using a solar charging system). Before 2015, devices were only rarely placed on subadults, and only if there remained unused GPS devices after sampling adults. After 2015, owing to increased funding, more sampling effort was dedicated to subadults.

Frank Marlowe and his students (B.W., C.B. and A.C.) collected additional GPS data with the Hadza between 2003 and 2006, and some of these data are reported in his book^23^. These data were available to us but by and large were not included in this study for technical reasons. Unfortunately, most of the GPS data from this period of study were saved in a file format that discarded time codes. Among those files that retained time codes, most did not cover a period of at least 8 hours. Five tracks collected before 2009, from the camps of Tuwa and Gangidape (Supplementary Table 2), met our inclusion criteria.

After collection, the GPS data were entered into a MySQL database. Spatial analyses of the GPS data were carried out in R, using custom software and existing spatial packages (sp, sf, maptools, raster, adehabitatHR and trip^67–72^). Each track was visually inspected using univariate (latitude or longitude) and bivariate (latitude and longitude) plots to identify errant GPS points. GPS data were also filtered by maximum movement speed and modal movement speed, allowing us to remove a few spatially errant points that had not been caught in prior filtering. Five person-days of data were excluded from analysis, because they record a day in which five participants were driven to an important village meeting using the research vehicle. We also removed from our dataset 12 additional person-days in which subjects hitched rides on motorcycles or bicycles. Thus, all the GPS data analysed here correspond to pedestrian travel.

All track points in the spatial database were categorized as ‘out of camp’ or ‘in camp’ based on whether they fell within the boundaries of the camp using maps made by researchers in each camp using a GPS device. Measures of distances travelled, steps walked and land explored are based on all travel. Measures of route sinuosity and interindividual proximity are based only on travel while out of camp, as explained in greater detail below.

A summary of the Hadza spatial database is provided in Supplementary Table 2.

Data collected from the camps of Tuwa in 2005, Gangidape in 2005 and Sengeli in 2011 are not included in the comparisons by gender of land areas visited or MCP size, because GPS data from these camps were extremely gender-imbalanced, including 100% female tracks, 100% male tracks and 97% female tracks, respectively.

When carrying out spatial analysis of GPS data, the temporal sampling rate of the GPS devices is critical to consider. During this study, the GPS devices were set to either record track points every 5 s (Canmore, BadElf) or use adaptive logging, which records more points when subjects are mobile and less when they are stationary (Garmin). In its raw form, the GPS data we collected had an average sampling rate of one reading per 8.3 s (s.d. = 8.0) and a median rate of 1 sample per 5 s. In order to make our data maximally equivalent across the study period and to enable comparisons with other populations, we interpolated all our GPS data prior to analysis so that it conformed to an even 5 s sampling interval. This interpolation has very little effect on the geometry of tracks, because the adaptive sampling system used in Garmin devices is designed not to lose information.

### General statistical strategy

Our data include repeated observations of individuals and locations and unequal sample sizes at these levels. Hierarchical modelling is useful for analysing such data because it prevents oversampled locations or individuals from excessively driving population estimates, and it allows one to estimate the influence of individual-level variation as well as variation at other levels, such as that structured by camp^73^. To implement these analyses, we use generalized additive mixed models developed using the R packages brms^74^ and mgcv^75^. When specifying a generalized additive mixed model, a predictor variable can be modelled in the same way as in a linear or generalized linear modelling context (for example, *y* ~ *x*), or it can be specified to relate to the dependent variable through a smooth function (for example, *y* ~ *S*(*x*)). Smooth functions allow for greater flexibility in the shape of estimated relationships between variables than is possible in linear modelling.

In this paper, we use smooth functions to estimate continuous relationships between age and measures of travel (models 1–3 and 7), days of observation and land explored (model 4) and distances from camp and proximity to others (model 7). The first goal of this paper is to describe trends in the data, and smooths are powerful, data-driven descriptive tools for such purposes^75,76^. Smooth functions are estimated using penalized thin plate regression^77^. The variance of the smooth function, which determines its ‘wiggliness’, is estimated from the data, like other parameters in the model. Using brms or mgcv, one must set the upper bound to this parameter by specifying a value for the ‘*k*’ parameter. This value determines the number of basis functions used to estimate smooths. If *k* is too low, a model may over-smooth relationships that exist in the data. After setting this upper bound, the actual smoothness of the resulting fit model is estimated from the data, conditional on this upper limit, using penalized thin plate regression. In our case, we left the ‘*k*’ parameter at its default value, which happens to be 10. To check the validity of this choice, we also fit models with *k* values at 11 and 12, and found that increasing *k* had no effect on the estimated smooths or the results. Smooth functions are useful for characterizing how physical or cognitive performance measures vary across age, because non-linear relationships between age and performance are often expected owing to normal patterns of growth and senescence. In the case of models 1–6 and 8, we fit the generalized additive mixed models using brms. In the case of model 7, owing to the very large size of the sociality sample, we were unable to fit the Bayesian model using brms. Instead we used the function ‘bam’ in the mgcv package, which is optimized for large datasets.

Priors used in the Bayesian models (1–6 and 8) were either vague or uniform, and each prior is listed in the detailed summary of statistical models in the Supplementary Information. Owing to the size of the spatial database, prior choice had little effect on our results. When generating posterior predictions from fit models, we control for the grouping factors of camp and individual by holding these random effects at their average values. This allows us to focus on the main variables of interest, which are age and gender.

In order to compare the predictive accuracy of our models to alternative model formulations, we also fit ‘reduced’ versions of models 1–7. The reduced versions of each model differ from the full versions in one respect: they do not include the main predictor variable of interest. For all models except model 3, this means that the reduced model does not include gender as a predictor variable. In the case of model 3, the reduced model does not include whether women were caring for a young dependent child in camp or not. For each model, we calculated information criteria to estimate the out-of-sample predictive accuracy of the full and reduced models. These information criteria are reported along with the detailed listing of statistical model formulae provided in the Supplementary Information.

### Distance walked

To estimate the relationship between age and distance walked per day, we fit a generalized additive mixed model (model 1). This model includes age as a smoothed predictor variable and gender as a covariate and includes varying intercept terms for the individual and camp observed. Smooth functions were estimated for each gender. Because geographic distances are bound to always be non-negative, we used a gamma distribution for the likelihood function. Both the shape and the scale of the distribution were specified to vary as a function of age, individual and camp.

A good reason for adopting a Bayesian approach is to be able to estimate not just the central tendency of outcome variables, but also the spread, or variance, of outcome variables, contingent on predictor variable values. Cohen’s *d* is a common measure of effect size used in studies of gender differences, and we wished to estimate Cohen’s *d* in distances walked per day across the range of ages in which we observed both genders studied (ages 3 to 64 years). However, calculating Cohen’s *d* directly from raw observational data that include repeated observations of individuals and camps would not be ideal. Thus, we computed Cohen’s *d* (and its distribution) from our fit model 1. First, we drew 1,000 samples from the posterior distribution of fit model 1, and for each sample, generated predictions for 1,000 days of male and female travel at each age and gender across the range of ages 3 to 64 years. After generating predictions for distances walked from the posterior of model 1, we then analysed these posterior predictions to compute Cohen’s *d* for each age in each sample from the posterior. The mean Cohen’s *d* value at each age and its 95% quantile CI are reported in Supplementary Table 5 and graphically represented in Extended Data Fig. 1.

To compare the daily travel of women with and without young children under their care, we first classified all women in the sample according to whether they had a co-resident child aged 2 years or younger under their care during our observations. The youngest age of a woman with such a child was 16 years, and the oldest was 45 years. Summary statistics describing this sample are listed in Supplementary Table 3.

We used this measure of child dependency as a jcategorical variable in a regression model predicting daily distances walked (model 3). After fitting the model, we then generated 1,000 predictions for daily travel across the range of ages for women with and without co-resident young children. The mean difference and the 95% quantile credible interval of this difference are plotted in Fig. 2. To compare the predictive accuracy of model 3 to a simpler model, we also fit a reduced null model which was the same in every other regard to model 3 but did not include the child-dependency predictor variable. A comparison of the WAIC values of these two models is reported in the main text and in the detailed listing of statistical models in the Supplementary Information.

### Steps per day

To estimate steps walked per day, we used data collected from Hadza research participants who wore both step-counting accelerometers and GPS devices for 123 observation days^44^. A multivariate Bayesian model was then fit to these data, in which stature, average movement speed and distance walked were used to predict recorded step counts. Here, we used the model trained on the Hadza data to generate step-count estimates for the full spatial database. For each person-day of data, we calculated the mean posterior prediction of step counts. We then fit a hierarchical model to step-count point estimates (model 2). The posterior predictions from model 2 are plotted in Fig. 1 across ages and were used to estimate the average steps per day reported in the main text for individuals aged 18–75 years.

The global step count data plotted in Fig. 1 were acquired from the GitHub repository associated with the study by Althoff et al.^45^ (https://github.com/timalthoff/activityinequality/blob/master/data/steps_by_age_gender_20170508.csv). Using these data, we calculated and report the mean steps of men and women by taking the simple (unweighted) mean across all age groups for each gender. This calculation matches the method used to calculate mean steps for the Hadza, which is similarly an unweighted mean.

### Land exploration

**Extended Data Fig. 3:**
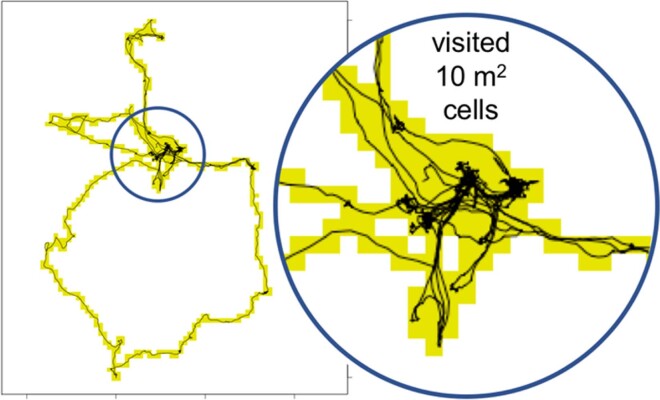
Raster measure of land visitation. Raster representation of land visitation covering one day of GPS data.

To measure how daily travel was distributed across landscapes, we constructed a raster representation of the landscapes surrounding each Hadza camp under study. In this raster model, each cell in the grid was 10 m × 10 m. We chose a cell size of 100 m^2^ because it appeared to be a reasonable approximation of a person’s ‘perception window’ while foraging. While arbitrary, fixing the cell size is necessary in order to make controlled comparisons. We then overlaid each GPS track upon these rasters and identified the cells each track intersected (Extended Data Fig. 3). The count of intersected cells formed the basis for two measures of land visitation.

Defining *A* as area in square metres of each raster cell (in our case, 100 m^2^), *N*_*i,i*_ as the number of unique cells visited on day *i* and *N*_*i,j*_ as the number of unique cells visited from days *i* through *j*, inclusive, we calculated the following measures at each day *i* for each person:

1. Daily land visited, *N*_*i,i*_ × *A*.
2. Cumulative land explored, *N*_1,*i*_ × *A*.

To help illustrate these measures, imagine a person who travelled 10 km on day 1, and their GPS track intersected 50 cells of our raster model. We would record that person as having (1) visited 5,000 m^2^ of land (50 cells × 100 m^2^) and (2) cumulatively explored 5,000 m^2^. Imagine now that on day 2, this same person travelled 15 km, and their track intersected 60 raster cells, 50 of which had not been visited on day 1. For day 2, we would calculate that this person had (1) visited 6,000 m^2^ of land and (2) cumulatively explored 10,000 m^2^ of land. We calculated these values for each day of GPS data in our database.

Our goal was to make a simple comparison between all men and women in terms of the cumulative land they explored across days of observation. In model 4, cumulative land explored is the dependent variable. Predictor variables include (smooth) days of observation (fit for each gender), and individual and camp as varying intercepts. Figure 3 displays the raw data of cumulative land exploration along with the estimated mean by gender and its 95% CI for average men and women.

**Extended Data Fig. 4:**
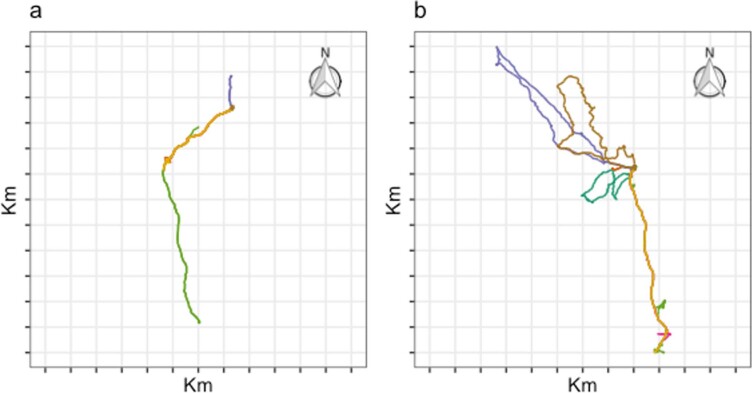
Gender-typical space use. One week of travel by the woman (**a**) and man (**b**) in our sample who rates of exploring land most closely matched gender averages. The tracks of the man in plot b intersected 7,188 10 m^2^ raster cells, while those of the woman in plot a intersected 2,780 cells. In addition to reaching more unique areas of the landscape, the travel of (**b**) was also more sinuous.

One week of GPS tracks from the individuals in our sample whose observed rates of land exploration most closely matched their gender’s average are displayed in Extended Data Fig. 4.

### Geographic segregation by gender

We used these same raster measures of landscape visitation to calculate geographic segregation between men and women. First, we calculated the total landscape area visited by all people in each camp, using raster union operations. Next, we calculated the landscape visited by men, that visited by women and the intersection of these areas, that is, the landscape visited by both genders studied. Table 1 summarizes these data in terms of cumulative land visited by all people in each camp, that visited by men, that visited by women and the extent of spatial overlap between male- and female-visited areas. The mean pattern of geographic segregation out of camp by gender across all camps is plotted in Fig. 4.

### MCP areas

In comparative research in movement ecology, ‘home range’ is often estimated and visualized by calculating MCPs that encompass locations visited^78^. Because MCPs are a fairly conventional way to characterize home ranges, we analysed our data in this manner. We calculated MCPs surrounding all male and female tracks in each camp, using the R package adehabitat^71^. Following convention, we calculated the MCPs to encompass 95% of the track points by camp and gender. Figure 5 and Supplementary Table 6 display and provide quantitative measures of MCPs in each camp.

### Sinuosity of travelled routes

Sinuosity (also called tortuosity), is defined as the ratio of an actual distance travelled between locations to the distance of the shortest as-the-crow-flies path. To measure how sinuous travelled paths were, we calculated for each track (1) outbound sinuosity, (2) inbound sinuosity and (3) geographically weighted sinuosity.

#### Outbound and inbound sinuosity

We first selected, from each track, those bouts of travel that represent the person leaving camp, travelling some distance on the landscape and then returning to camp. Consistent with our prior work^20^, we only analysed those bouts where individuals travelled at least 500 m from camp. If there was more than one such bout in a day, we selected the longest-distance bout for analysis. We selected the point where they left camp, the furthest point on the landscape that they reached (in terms of distance from the camp centroid) and the point where they returned to camp. We used the furthest point they reached to split this bout into an outbound segment and an inbound segment. For each segment, we calculated both the path length as travelled and the as-the-crow-flies shortest path length between the segment’s start and end points. We then divided the actual path distances by their respective shortest path lengths, resulting in measures of outbound and inbound sinuosity. It was possible to estimate inbound and outbound sinuosity measures for 1,526 tracks in our sample.

To compare the sinuosity of male and female travel, we first performed nonparametric Mann–Whitney tests on the raw data. Next, we fit generalized additive mixed models to outbound (model 5) and inbound (model 6) sinuosity. Prior to fitting the models, sinuosity was standardized to aid model convergence. In these models, standardized sinuosity is the outcome variable, while gender is a linear fixed effect and individual and camp are random effects (varying intercepts). Bivariate plots of the raw data showed no relationships between age or track length and sinuosity, so these were not included in the models. One thousand samples from the posterior distributions of the fit models were used to estimate patterns by gender.

#### Geographically weighted sinuosity

Geographically weighted sinuosity measures over how many geographically distinct places people engaged in sinuous travel. We developed this measure to distinguish between a route that includes a series of turns taking place near one another from a route that includes an equal number of turns distributed across the landscape. We surmise that a series of geographically distinct reorientations would be more navigationally challenging. To measure geographically distinct bouts of turning, for each track, we segmented the total out-of-camp travel by overlaying a grid of 10 m × 10 m cells upon the track, and then calculated the average sinuosity of all travel that crossed through each cell. Travel through a particular cell was scored as ‘high sinuosity’ if its sinuosity value was higher than the population average (from all cells in all tracks) and scored as ‘very high sinuosity’ if its sinuosity was higher than 99% of all cells in the total sample. For each track, we then counted these scores. We plot the distribution of these geographically weighted sinuosity scores for all tracks by gender in Extended Data Fig. 2.

### Sociality while travelling out of camp

**Extended Data Fig. 5:**
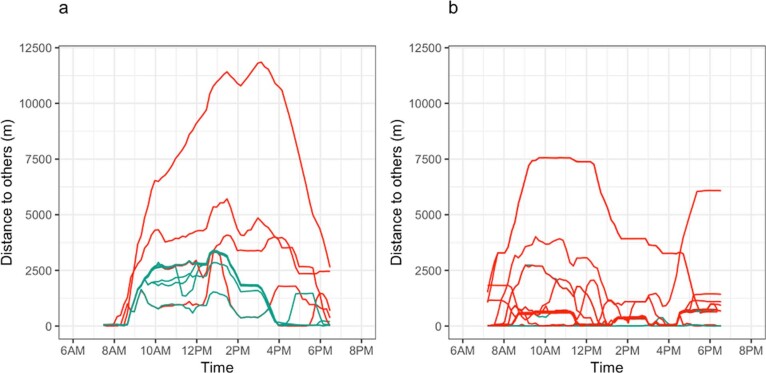
GPS measures of physical proximity. Proximities to others throughout the day for a 33-year-old man (**a**) and a 31-year-old woman (**b**). Proximities to women and girls are plotted in green, and to men and boys, red.

The sociality sample consists of 197,244 samples of the physical proximities of all camp members wearing GPS devices. To construct this sociality sample, we drew a random sample of 100 out-of-camp track points from each person-day of GPS data. If a total of 100 track points were not available from a given track, the sample includes all of the out of camp track points recorded from that track. For each track point in the sociality sample, we computed the straight-line distances between the sampled individual and all others with recent (within ±60 s) GPS measures. To help illustrate these measures, we plot in Extended Data Fig. 5 the physical proximities of one man and one woman to all others with GPS data throughout a day.

Across the entire sociality sample, we calculated whether the target individual was within 5 m of their nearest neighbour. To test the hypothesis that women are more social than men while out of camp, we report in Supplementary Table 4 the median distance from women and men to their nearest neighbours across the entire sociality sample and within seasonal subsamples.

To explore how physical proximities between individuals differed by gender and distance from camp, we fit generalized additive mixed models to the sociality sample. In model 7, the dependent variable was the binary measure ‘is within 5 m of a campmate’. This model also included the following predictor variables: age of target individual (smoothed), the target individual’s distance from camp (smoothed), the number of other individuals with recent GPS measures, and the following two grouping factors: the individual (random intercept) and the camp (random intercept).

Owing to the very large size of the sociality sample, we were unable to fit model 7 using the Bayesian methods in the package brms. Instead, we used the function ‘bam’ available in the mgcv package^75^, which is optimized for very large datasets. The smoothing parameter that controls the degree of wiggliness in the smooth function was estimated using the maximum likelihood method.

Using the fit model 7 we plot the model-predicted probability of an average man or woman being in close proximity to a campmate as a function of their distance from camp (Fig. 7). In this analysis, we statistically controlled for the influences of age, the number of people wearing GPS devices and random effects at the level of individual and camp by holding them all at their mean values.

### Reporting Summary

Further information on research design is available in the Nature Research Reporting Summary linked to this article.

## Supplementary information

Supplementary Information Supplementary Figs. 1–3, Supplementary Tables 1–6, Supplementary Methods and Supplementary Results.

Reporting Summary

## Data Availability

Owing to privacy concerns, the raw GPS data underlying our analyses are not shared publicly. Please contact the corresponding author to access the data and discuss terms for ethical use.

## Code availability

The R code used to fit all statistical models is provided in the Supplementary Information. The R code used to estimate step counts from GPS data is described by Wood et al.^44^ and available at the Open Science Foundation at https://osf.io/5z8q2. Other custom code that supports the findings of this study is available from the corresponding author upon request.
