# Supplementary material for: Gendered movement ecology and landscape use in Hadza hunter-gatherers
Source: Nat Hum Behav. 2021 Jan 4;5(4):436–46. doi: 10.1038/s41562-020-01002-7 (PMC8060163; doi:10.1038/s41562-020-01002-7)
Supplement: Supplementary file 1 — Supplementary Figs. 1–3, Supplementary Tables 1–6, Supplementary Methods and Supplementary Results. [file 41562_2020_1002_MOESM1_ESM.pdf]

nature research https://doi.org/10.1038/s41562-020-01002-7

Supplementary information

Gendered movement ecology and landscape use in Hadza hunter-gatherers

In the format provided by the authors and unedited

SUPPLEMENTARY METHODS

Research participants

Gender	N participants	Mean age	SD age
Male	92	34.5	17.4
Female	87	37.0	20.7
All	179	35.8	19.2

Supplementary Table 1. Age and number of research participants in this study

Hadza camps in which data were collected

Camp	N female tracks	N male tracks	N tracks	Year
Tuwa	2	0	2	2005
Gangidape	0	3	3	2005
Setako	59	45	104	2009
Setako	50	32	82	2010
Sengeli	42	48	90	2010
Sengeli	109	4	113	2011
Sanola	108	95	203	2014
Buruku	93	62	155	2015
Sengeli	68	72	140	2015
Kideru Juu	58	112	170	2016
Hukumako	223	226	449	2016
Buruku	86	83	169	2016
Hukumako	66	40	106	2017
Ol Piro	77	116	193	2018
Hukumako	56	43	99	2018
Sum	1097	981	2078	

Supplementary Table 2. Counts of GPS tracks in our spatial database by camp and gender.

Sample of women used to compare daily travel by child dependency

Status	N individuals	N tracks	Mean age (SD)
Female co-resident with child aged 2 or younger	19	244	28.0 (9.3)
Female aged 16-45, without co-resident young child	39	434	32.4 (9.1)

Supplementary Table 3. The sample of women used to examine the impact of young children on their mothers’ day range.

Details of statistical models

Model 1: Distance walked per day by age and gender

brms formula: bf(track_length_km ~ track_subject_gender + s(track_subject_age, by = track_subject_gender) + (1 | fk_person_id) + (1 | fk_camp))

Likelihood: Gamma

Link function: Log

Priors:

- Intercept ~ uniform(1, 3)
- Intercept_shape ~ student_t(3, 0, 10)
- All regression coefficients: normal(0,100)
- sd ~ student_t(3, 0, 10)
- sd_shape ~ student_t(3, 0, 10)
- sds ~ normal(0, 100)
- sds_shape ~ student_t(3, 0, 10)

Model 1 WAIC: 12225.5

Reduced model 1 without gender term WAIC: 12238.8

Model 2: Steps per day by age and gender

Likelihood: Gamma

Link function: Log

brms formula: bf(step_count_estimate ~ track_subject_gender + s(track_subject_age, by=track_subject_gender) + (1|fk_person_id) + (1|fk_camp), shape~track_subject_gender + s(track_subject_age, by=track_subject_gender) + (1|fk_person_id) + (1|fk_camp))

Priors:

- Intercept ~ student_t(3, 9, 10)
- Intercept_shape ~ student_t(3, 0, 10)
- All regression coefficients: normal(0,1000)
- sd ~ student_t(3, 0, 10)
- sd_shape ~ student_t(3, 0, 10)
- sds ~ normal(0, 100)

sds_shape ~ student_t(3, 0, 10)

Model 2 WAIC: 42665.9

Reduced Model 2 without gender term: 42678.5

Model 3: Female distance walked per day by age and child dependency

brms formula: bf(track_length_km ~ has_child_2_or_younger + s(track_subject_gender, by=has_child_2_or_younger) + (1|fk_person_id) + (1|fk_camp), shape~has_child_2_or_younger+ s(track_subject_age, by=has_child_2_or_younger) + (1|fk_person_id) + (1|fk_camp))

Likelihood: Gamma

Link function: Log

Priors:

Intercept ~ student_t(3, 2, 10)

Intercept_shape ~ student_t(3, 0, 10)

All regression coefficients: normal(0,10)

sd ~ student_t(3, 0, 10)

sd_shape ~ student_t(3, 0, 10)

sds ~ normal(0, 100)

sds_shape ~ student_t(3, 0, 10)

M3 WAIC: 3828.2

Reduced M3 without child dependency term: 3823.4

Model 4: Cumulative land explored by gender and days of observation

brms formula: bf(cummulative_area_visited_km2 ~ track_subject_gender + s(day_number, by=track_subject_gender) + (1|fk_camp) + (1|fk_person_id), sigma~ track_subject_gender + s(day_number, by=track_subject_gender) + (1|fk_camp) + (1|fk_person_id))

Likelihood: Gaussian

Model 4 WAIC: -3881.3

Reduced model 4 WAIC without gender term: -3035.0

Model 5: Outbound sinuosity by gender

brms formula: bf(outbound_sinuosity_standardized ~ track_subject_gender + (1|fk_person_id) + (1|fk_camp), sigma ~ track_subject_gender + (1|fk_person_id)+ (1|fk_camp))

Likelihood: Gaussian

Priors:

Intercept ~ normal(0,1)

Intercept_sigma ~ normal(1, 1)

All regression coefficients: normal(0,1)

sd ~ normal(0, 1)

sd_sigma ~ student_t(3, 0, 10)

Model 5 WAIC: 3655.2

Reduced model 5 without gender WAIC: 3666.8

Model 6: Inbound sinuosity by gender

brms formula: bf(inbound_sinuosity_standardized ~ track_subject_gender + (1|fk_person_id) + (1|fk_camp), sigma ~ track_subject_gender + (1|fk_person_id)+ (1|fk_camp))

Priors:

Intercept ~ normal(0,1)

Intercept_sigma ~ normal(1, 1)

All regression coefficients: normal(0,1)

sd ~ normal(0, 1)

sd_sigma ~ student_t(3, 0, 10)

Model 6 WAIC: 3563.3

Reduced model 6 without gender WAIC: 3562.9

Model 7: Sociality out of camp by gender and age

mgcv formula:
bam(campmate_within_5_m~n_other_people_wearing_GPS+gender_subject+s(age_subject, by=gender_subject)+s(distance_from_camp_subject, by=gender_subject) + s(person_id_subject, bs="re") + s(fk_camp, bs="re"), family=binomial, data=data, method=”ML”)

Model 7 AIC: 221886.6

Reduced model 7 without gender AIC: 223315.3

Model 8: Distance walked per day by age, gender, and season

brms formula: bf(track_length_km ~ track_subject_gender + is_dry_season + s(track_subject_age, by = track_subject_gender) + (1 | fk_person_id) + (1 | fk_camp))

Likelihood: Gamma

Link function: Log

Priors:

Intercept ~ uniform(1, 3)

Intercept_shape ~ student_t(3, 0, 10)

All regression coefficients: normal(0,100)

sd ~ student_t(3, 0, 10)

sd_shape ~ student_t(3, 0, 10)

sds ~ normal(0, 100)

sds_shape ~ student_t(3, 0, 10)

Model 8 WAIC: 12226.8

Reduced model 8 without season term: see model 1.

SUPPLEMENTARY RESULTS

Proximities of individuals by season and gender in the sociality sample

Gender of target individual	Season	Median distance to nearest neighbor meters (25%, 75% quantile)	N target individuals	Mean age target (SD)	N days	N samples	Mean simultaneous GPS per sample (SD)
Female	Dry	3.4 (1.4, 21)	70	37.2 (21.6)	135	76489	13.4 (5.3)
Female	Wet	2.6 (1.1, 23.2)	40	36 (16.7)	34	22715	13.7 (4.9)
Female	All	3.2 (1.3, 21.5)	85	36.9 (20.6)	169	99204	13.4 (5.2)
Male	Dry	55.1 (3, 891)	76	32.3 (16.9)	134	77507	12.9 (5.8)
Male	Wet	103.1 (2.5, 977)	38	42.2 (15.8)	25	16533	15.3 (4.8)
Male	All	63.3 (2.9, 905.2)	93	34.1 (17.2)	159	94040	13.4 (5.7)
All	All	5.9 (1.8, 231.2)	178	35.5 (19)	172	193244	13.4 (5.4)

Supplementary Table 4. Summary statistics of the sociality sample. Distances between target individuals and all other campmates were computed, and the distance to their nearest neighbor determined. The mean number of individuals simultaneously wearing GPS devices across the sample was 13.4, which is approximately half the population of a typical Hadza camp.

Distances walked per day

Below we tabulate the mean distances walked per day by gender and age, and the resulting gender differences in terms of Cohen's D. The values for each age were computed from 1000 simulations from the posterior distribution of fit model 1. In each simulation, 1000 days of male and female travel at each age were predicted from the posterior distribution. Random effects at the individual and camp-level were held at their average values. The measure of gender difference, Cohen's D, is the difference of means divided by the pooled standard deviation. Positive values represent further distance travel by males.

Age	Male mean	Female mean	Pooled SD	Cohen's D (95% quantile)
3	5.32	5.7	1.82	-0.21 (-0.3, -0.12)
4	5.83	6.03	1.95	-0.1 (-0.19, -0.02)
5	6.36	6.37	2.11	0 (-0.08, 0.08)
6	6.94	6.71	2.29	0.1 (0.02, 0.18)
7	7.55	7.07	2.51	0.19 (0.11, 0.28)
8	8.18	7.41	2.75	0.28 (0.19, 0.36)
9	8.82	7.74	3.02	0.36 (0.26, 0.44)
10	9.44	8.03	3.3	0.43 (0.34, 0.51)
11	10.04	8.3	3.59	0.48 (0.39, 0.57)
12	10.61	8.52	3.88	0.54 (0.46, 0.62)
13	11.13	8.71	4.16	0.58 (0.5, 0.66)
14	11.6	8.85	4.44	0.62 (0.54, 0.7)
15	12.01	8.95	4.71	0.65 (0.57, 0.74)
16	12.38	9.02	4.97	0.68 (0.6, 0.76)
17	12.7	9.05	5.21	0.7 (0.62, 0.78)
18	12.97	9.07	5.43	0.72 (0.63, 0.8)
19	13.2	9.05	5.65	0.74 (0.65, 0.82)
20	13.42	9.03	5.83	0.75 (0.66, 0.83)
21	13.61	9	6.01	0.77 (0.69, 0.85)
22	13.8	8.96	6.14	0.79 (0.7, 0.87)
23	13.98	8.92	6.28	0.81 (0.72, 0.89)
24	14.14	8.87	6.38	0.83 (0.75, 0.91)
25	14.3	8.8	6.47	0.85 (0.77, 0.94)

26	14.45	8.74	6.55	0.87 (0.79, 0.95)
27	14.61	8.67	6.62	0.9 (0.82, 0.98)
28	14.73	8.59	6.68	0.92 (0.84, 1)
29	14.85	8.49	6.72	0.95 (0.87, 1.03)
30	14.93	8.39	6.74	0.97 (0.89, 1.05)
31	14.98	8.28	6.74	0.99 (0.91, 1.08)
32	15	8.17	6.75	1.01 (0.94, 1.09)
33	15.01	8.04	6.75	1.03 (0.95, 1.11)
34	14.95	7.93	6.72	1.04 (0.96, 1.13)
35	14.91	7.82	6.71	1.06 (0.97, 1.14)
36	14.78	7.71	6.69	1.06 (0.98, 1.14)
37	14.65	7.61	6.65	1.06 (0.98, 1.14)
38	14.52	7.51	6.62	1.06 (0.97, 1.14)
39	14.35	7.45	6.6	1.05 (0.97, 1.12)
40	14.17	7.38	6.56	1.04 (0.96, 1.12)
41	13.99	7.33	6.53	1.02 (0.94, 1.1)
42	13.82	7.3	6.51	1 (0.92, 1.08)
43	13.65	7.28	6.49	0.98 (0.9, 1.06)
44	13.5	7.28	6.5	0.96 (0.88, 1.04)
45	13.35	7.28	6.5	0.93 (0.85, 1.01)
46	13.22	7.3	6.52	0.91 (0.82, 0.99)
47	13.09	7.32	6.54	0.88 (0.8, 0.96)
48	12.99	7.33	6.57	0.86 (0.78, 0.94)
49	12.89	7.35	6.6	0.84 (0.76, 0.92)
50	12.77	7.38	6.62	0.81 (0.73, 0.89)
51	12.71	7.39	6.67	0.8 (0.72, 0.87)
52	12.61	7.41	6.7	0.78 (0.7, 0.85)
53	12.5	7.41	6.71	0.76 (0.68, 0.83)
54	12.41	7.41	6.75	0.74 (0.66, 0.82)
55	12.25	7.39	6.72	0.72 (0.64, 0.8)
56	12.1	7.37	6.71	0.7 (0.63, 0.78)
57	11.92	7.35	6.68	0.68 (0.61, 0.76)
58	11.68	7.31	6.61	0.66 (0.59, 0.74)
59	11.45	7.26	6.52	0.64 (0.57, 0.72)
60	11.15	7.21	6.41	0.61 (0.54, 0.69)
61	10.82	7.15	6.28	0.58 (0.51, 0.66)
62	10.46	7.08	6.14	0.55 (0.47, 0.63)
63	10.08	7	5.96	0.52 (0.43, 0.6)
64	9.65	6.91	5.79	0.47 (0.4, 0.55)
65	9.22	6.82	5.59	0.43 (0.35, 0.51)
66	8.79	6.72	5.41	0.38 (0.3, 0.46)
67	8.35	6.6	5.24	0.33 (0.25, 0.41)
68	7.95	6.49	5.08	0.29 (0.21, 0.37)

Supplementary Table 5. Mean, pooled standard deviation, and gender difference (D) of distances walked by age.

The values in Supplementary Table 5 show that around age 6, males traveled further than females on average. At age 6, this difference amounts to a D value of 0.10 (0.02, 0.18), which is a modest difference. The gender difference is more pronounced at age 10 (D=0.43 [0.34, 0.51]) and continues to climb, and is at its highest across ages 30-45, during which male travel distances are 1 standard deviation higher than female distances (D ~ 0.93-1.06). After the mid-40s, the gender difference declines, largely owing to decreasing travel by men (Extended Data Figure 3).

Land explored

Camp	Year	Male MCP km^2	Female MCP km^2	MM:FF ratio
Setako	2009	59	23	2.6
Setako	2010	84	9	9.5
Sengeli	2010	89	8	10.9
Sanola	2014	82	18	4.4
Sengeli	2015	88	25	3.5
Buruku	2015	47	10	4.9
Buruku	2016	131	14	9.2
Hukumako	2016	135	32	4.2
Kideru Juu	2016	16	13	1.2
Hukumako	2017	47	11	4.2
Ol Piro	2018	56	10	5.4
Hukumako	2018	47	46	1.0
Mean		73	18	4.0

Supplementary Table 6. The area of male and female minimum convex polygons in each camp.

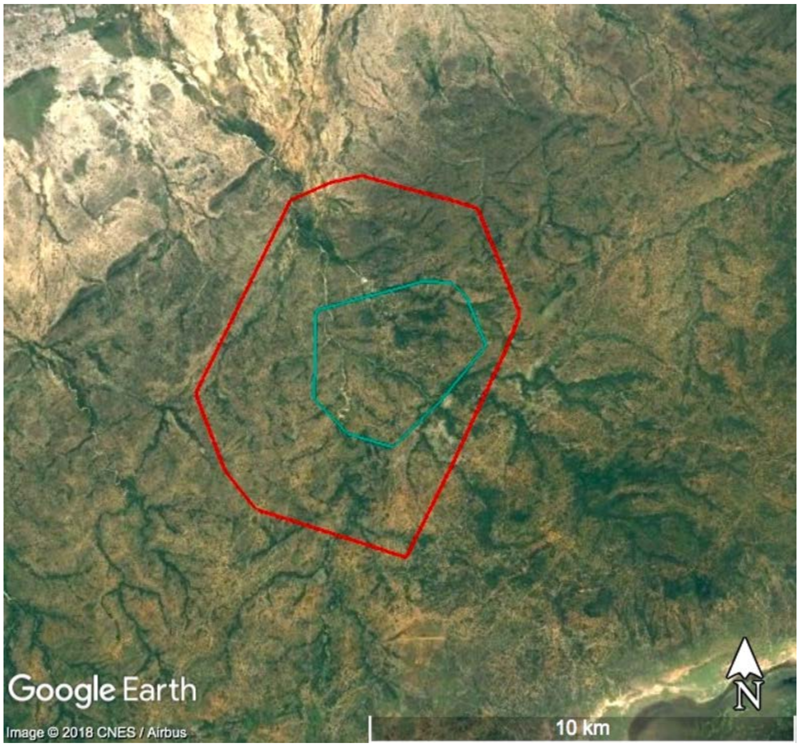

Supplementary Information Figure 1. Minimum convex polygons surrounding male (in red) and female (in green) tracks from a typical camp -- Hukumako in 2017. Map data: Google, CNES/Airbus

Camp-level and seasonal variation in spatial behavior

To place this study in context it is useful to get a broad sense of differences in spatial behavior across camps. Below, we plot the empirical mean distance traveled by men and women aged 20 to 39 across all camps.

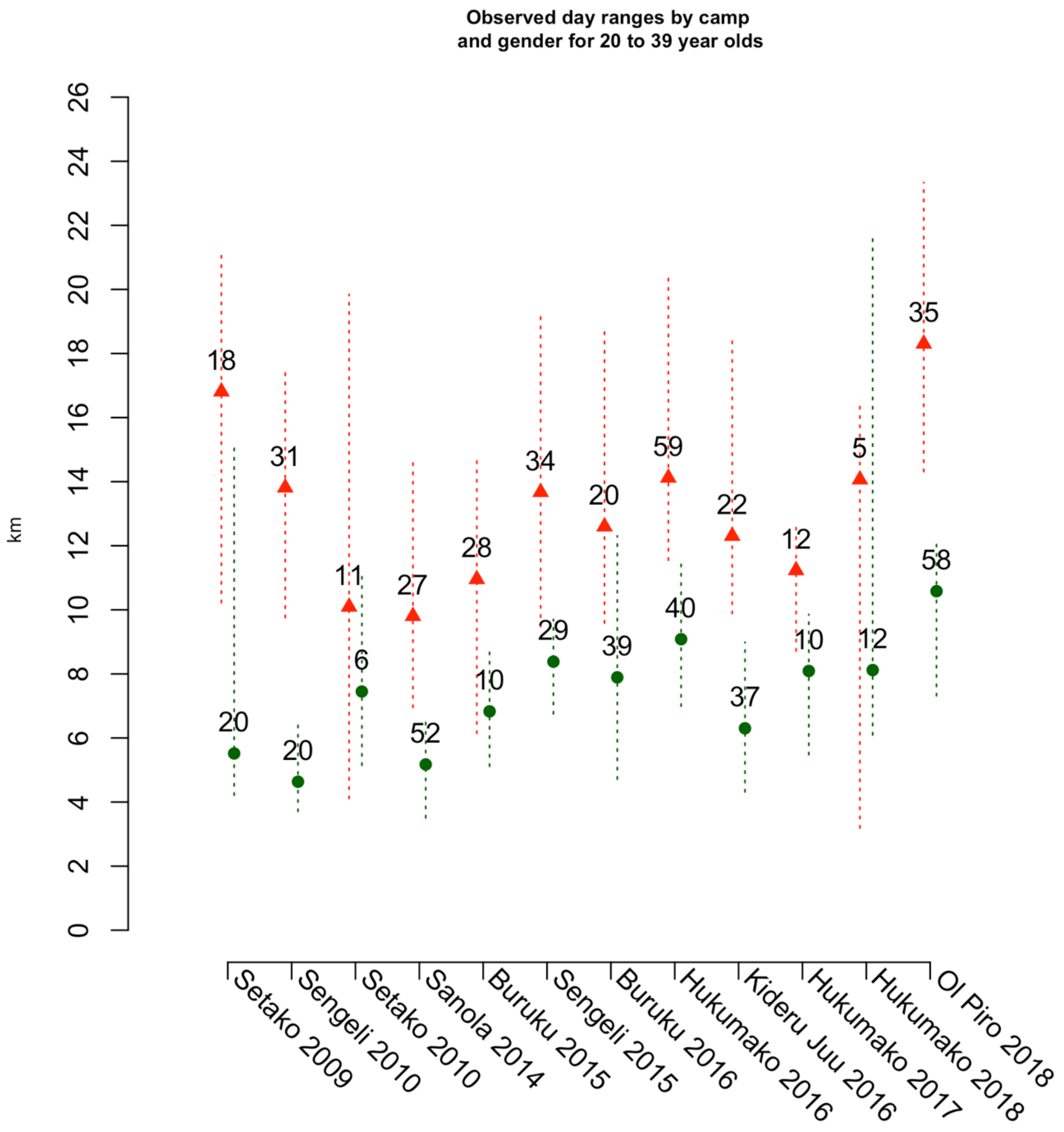

Supplementary Information Figure 2. Distances walked per day, summarized by gender and camp among those aged 20 to 39. Red arrows represent men's median values, green circles represent women's median values. The dotted lines extend across the interquartile ranges of each camp-gender group. The number of GPS tracks in each group is plotted.

As seen in Supplementary Figure 2, the raw empirical median distance traveled by men was higher than women in 12/12 camps under study. In Hukumako 2018, the interquartile ranges of distances

walked by gender overlap considerably. As is seen in Figure 5, the minimum convex polygons of male and female “home ranges” in this camp were also quite similar. Inspection of the GPS tracks and field notes of that camp indicates that this pattern arose owing to several days during which people left in mixed-gender groups for a combination of foraging activities and social visits to neighboring Hadza camps.

We also examined the role of seasonal differences and assessed whether a variable coding for season (dry season: June-October, wet season: November-May) would contain predictive information about day range (model 8) or physical proximities between individuals in the sociality sample. Season was not associated with a difference in day range and the addition of a season variable in model 8 led to a decrease in estimated out of sample predictive accuracy (model 8 WAIC=12226.8 vs model 1 WAIC=12225.5). Similarly, in the sociality sample the distance between target individuals and their nearest neighbors did not vary meaningfully as a function of season (see Supplementary Table 4).

Visualization of male and female spatial behavior

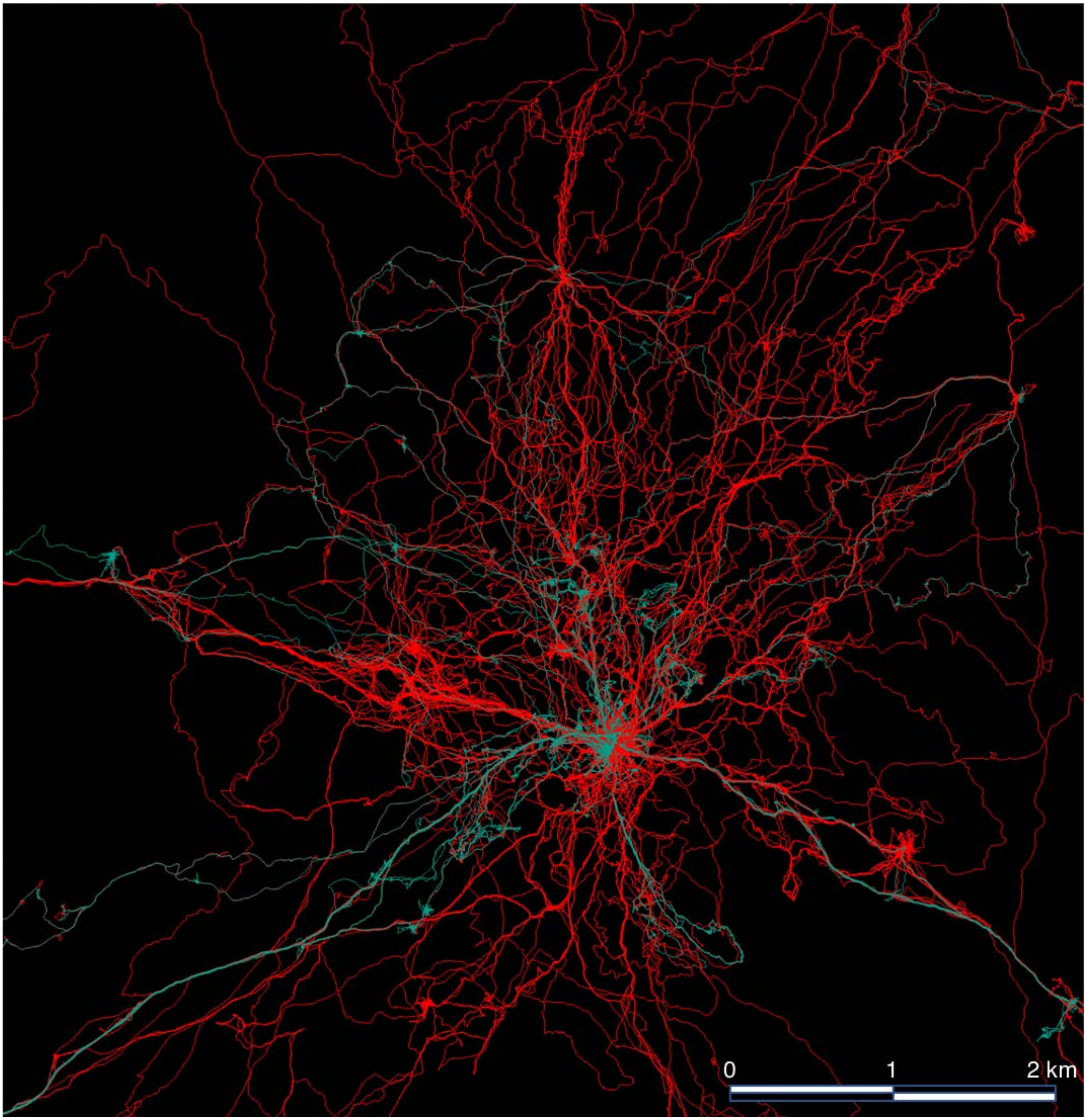

Supplementary Information Figure 3. Overview of male (red) and female (green) GPS tracks recorded in the camp of Hukumako in 2016. Note the more extensive travel by males, and the rarity of mixed-gender travel outside of camp.
