# Supplementary material for: Gendered movement ecology and landscape use in Hadza hunter-gatherers
Source: Nat Hum Behav. 2021 Jan 4;5(4):436–46. doi: 10.1038/s41562-020-01002-7 (PMC8060163; doi:10.1038/s41562-020-01002-7)
Supplement: Supplementary file 2 — Reporting Summary [file 41562_2020_1002_MOESM2_ESM.pdf]

nature research

Corresponding author(s): Brian Wood

Last updated by author(s): 21-Aug-2020

Reporting Summary

Nature Research wishes to improve the reproducibility of the work that we publish. This form provides structure for consistency and transparency in reporting. For further information on Nature Research policies, see our Editorial Policies and the Editorial Policy Checklist.

Statistics

For all statistical analyses, confirm that the following items are present in the figure legend, table legend, main text, or Methods section.

n/a	Confirmed	
☐	☒	The exact sample size (*n*) for each experimental group/condition, given as a discrete number and unit of measurement
☐	☒	A statement on whether measurements were taken from distinct samples or whether the same sample was measured repeatedly
☐	☒	The statistical test(s) used AND whether they are one- or two-sided<br>*Only common tests should be described solely by name; describe more complex techniques in the Methods section.*
☐	☒	A description of all covariates tested
☐	☒	A description of any assumptions or corrections, such as tests of normality and adjustment for multiple comparisons
☐	☒	A full description of the statistical parameters including central tendency (e.g. means) or other basic estimates (e.g. regression coefficient) AND variation (e.g. standard deviation) or associated estimates of uncertainty (e.g. confidence intervals)
☐	☒	For null hypothesis testing, the test statistic (e.g. *F*, *t*, *r*) with confidence intervals, effect sizes, degrees of freedom and *P* value noted<br>*Give P values as exact values whenever suitable.*
☐	☒	For Bayesian analysis, information on the choice of priors and Markov chain Monte Carlo settings
☐	☒	For hierarchical and complex designs, identification of the appropriate level for tests and full reporting of outcomes
☐	☒	Estimates of effect sizes (e.g. Cohen's *d*, Pearson's *r*), indicating how they were calculated

Our web collection on statistics for biologists contains articles on many of the points above.

Software and code

Policy information about availability of computer code

Data collection	*Provide a description of all commercial, open source and custom code used to collect the data in this study, specifying the version used OR state that no software was used.*
Data analysis	Analysis was conducted using R version 3.5.3. The R code used to fit all statistical models is shared in the supplementary materials. The R code used to estimate step counts from GPS data is available at the Open Science Foundation at https://osf.io/5z8q2. Other custom code that supports the findings of this study is available from the corresponding author upon request.

For manuscripts utilizing custom algorithms or software that are central to the research but not yet described in published literature, software must be made available to editors and reviewers. We strongly encourage code deposition in a community repository (e.g. GitHub). See the Nature Research guidelines for submitting code & software for further information.

Data

Policy information about availability of data

All manuscripts must include a data availability statement. This statement should provide the following information, where applicable:

- Accession codes, unique identifiers, or web links for publicly available datasets
- A list of figures that have associated raw data
- A description of any restrictions on data availability

Owing to privacy concerns, the GPS data underlying our analyses are not shared publicly. Please contact the corresponding author to access the data and discuss terms for ethical use.

Field-specific reporting

Please select the one below that is the best fit for your research. If you are not sure, read the appropriate sections before making your selection.

☐ Life sciences ☒ Behavioural & social sciences ☐ Ecological, evolutionary & environmental sciences

For a reference copy of the document with all sections, see nature.com/documents/nr-reporting-summary-flat.pdf

Behavioural & social sciences study design

All studies must disclose on these points even when the disclosure is negative.

Study description	Observational research aided by lightweight sensors. Mixed methods research including quantitative and qualitative measures.
Research sample	Hadza hunter-gatherers living in northern Tanzania in the Lake Eyasi region
Sampling strategy	Randomized. Overall, we sought to capture as many GPS samples as time and resources and field logistics permitted. We arbitrarily decided to cease data collection for this study after we had amassed more than 2,000 GPS tracks. This was deemed satisfactory not based on a statistical power analysis, but by the heuristic that this was a far larger sample of GPS tracks than had ever been collected for a study of daily spatial behavior in a traditional population.
Data collection	The total dataset includes 2078 person-days (23,872 person-hours) of GPS measured movement. This includes 1097 person-days of female movement and 981 person-days of male movement. GPS data were collected by asking Hadza in 15 camps to wear small GPS devices (manufactured by Garmin, BadElf, and Canmore). We continued GPS data collection for this study until we reached an arbitrary target of at least 2000 total GPS tracks, a sample size substantially higher than acquired for any published study of human movement in a traditional society. During our data collection, we endeavored to place GPS devices on a random sample of adult camp members each day. One or two researchers would walk through camp early in the morning as people were rousing. We would greet people at their homes or hearths, and hand out GPS devices to be worn during the day. We varied the path we would walk through the camps so as to randomize which individuals would wear devices throughout the study. The devices were worn during the daylight hours, typically attached to belts or upper arms. Devices were likewise removed by researchers around nightfall, when most people had returned to camp and were retiring for the evening. In the analysis reported here, we only include data from subjects whose GPS devices recorded data for at least eight hours. The criterion of a minimum of eight hours allows for meaningful comparisons of spatial behavior across the normally physically active hours of the day. In this sample of 2078 tracks, the GPS devices recorded data for 11.49 hours on average (SD=2.04). Data collection and analysis were not performed blind to the hypotheses of the study. On average, devices were put on at 07:45:52 and stopped recording at 18:57:53. On some occasions, individuals did not return to camp by the early evening, but stayed out of camp, usually to hunt at night or to visit a neighboring Hadza settlement. In these cases, the GPS devices were not collected until their wearers returned (usually the next morning). The total number of GPS devices used each day varied throughout the study, as a function of funding (how many we could purchase for a field season) and weather (how many devices we could charge using a solar charging system). Before 2015, devices were rarely placed on sub-adults, and only after sampling adults, if there remained unused GPS devices. After 2015, owing to increased funding, more sampling effort was dedicated to sub-adults.
Timing	5/12/05<br>5/17/05<br>5/22/05<br>12/2/05<br>12/3/05<br>8/27/09<br>8/28/09<br>8/29/09<br>8/30/09<br>8/31/09<br>9/1/09<br>9/2/09<br>9/3/09<br>9/4/09<br>9/5/09<br>9/6/09<br>9/7/09<br>9/8/09<br>5/29/10<br>5/30/10<br>5/31/10<br>6/1/10<br>6/2/10<br>6/3/10<br>6/4/10<br>6/5/10<br>6/6/10<br>6/7/10<br>6/14/10

6/15/10
6/16/10
6/17/10
6/18/10
6/19/10
6/20/10
6/21/10
6/22/10
6/23/10
6/24/10
1/5/11
1/6/11
1/7/11
1/8/11
1/9/11
1/10/11
1/11/11
1/12/11
1/13/11
1/15/11
1/16/11
8/13/14
8/14/14
8/15/14
8/16/14
8/17/14
8/18/14
8/19/14
8/20/14
8/21/14
8/22/14
8/23/14
8/24/14
8/25/14
8/26/14
8/27/14
8/28/14
8/29/14
8/30/14
8/31/14
9/1/14
9/2/14
7/3/15
7/4/15
7/5/15
7/6/15
7/7/15
7/8/15
7/9/15
7/10/15
7/14/15
7/25/15
7/26/15
7/27/15
7/28/15
7/29/15
7/30/15
7/31/15
8/1/15
8/2/15
8/3/15
8/5/15
8/6/15
8/7/15
8/8/15
8/9/15
8/11/16
8/12/16
8/13/16
8/14/16
8/15/16
8/16/16
8/17/16
8/18/16
8/19/16
8/20/16

8/21/16
8/22/16
8/23/16
8/24/16
8/25/16
8/26/16
8/27/16
8/28/16
8/29/16
8/30/16
8/31/16
9/1/16
9/2/16
9/4/16
9/5/16
9/6/16
9/10/16
9/11/16
9/12/16
9/13/16
9/14/16
9/15/16
9/16/16
9/17/16
9/18/16
9/19/16
9/20/16
9/21/16
10/7/16
10/8/16
10/9/16
10/10/16
10/11/16
10/12/16
10/13/16
10/14/16
10/15/16
10/16/16
10/17/16
10/18/16
10/19/16
10/20/16
10/21/16
10/22/16
10/23/16
10/24/16
6/17/17
6/18/17
6/19/17
6/20/17
6/21/17
6/23/17
2/11/18
2/12/18
2/13/18
2/14/18
2/15/18
2/16/18
2/27/18
2/28/18
3/1/18
3/2/18
3/3/18
3/4/18
3/5/18
3/6/18
3/7/18
3/8/18
3/9/18
3/10/18
3/11/18
3/12/18

Data exclusions

Five person-days of data were excluded from analysis, because they record a day in which five participants were driven to an important village meeting using the research vehicle. We also removed from our dataset 12 additional person-days in which subjects

	hitched rides on motorcycles or bicycles. Thus, all the GPS data analyzed here corresponds to pedestrian travel.
Non-participation	No participants declined
Randomization	Data collection was randomized as described in the data collection section. The analyses were carried out using hierarchical models which allow us to statistically adjust estimates for random effects and other co-variates. All of our statistical adjustments or "controls" are described in detail in the manuscript.

Reporting for specific materials, systems and methods

We require information from authors about some types of materials, experimental systems and methods used in many studies. Here, indicate whether each material, system or method listed is relevant to your study. If you are not sure if a list item applies to your research, read the appropriate section before selecting a response.

Materials & experimental systems

n/a	Involved in the study
☒	☐ Antibodies
☒	☐ Eukaryotic cell lines
☒	☐ Palaeontology and archaeology
☒	☐ Animals and other organisms
☐	☒ Human research participants
☒	☐ Clinical data
☒	☐ Dual use research of concern

Methods

n/a	Involved in the study
☒	☐ ChIP-seq
☒	☐ Flow cytometry
☒	☐ MRI-based neuroimaging

Human research participants

Policy information about studies involving human research participants

Population characteristics	Hadza hunter-gatherers of northern Tanzania
Recruitment	During the fieldwork for this research, we sought out those Hadza camps where people were subsisting from hunting and gathering for the vast majority of their diet. We did not carry out work among camps where wage labor or tourism was occurring. Using scripts approved by the ethical review boards of our institutions (listed below) we described our research interests, the daily activities our research would entail, the duration of our research, and the payments in the form of gifts that would be given at the close of our research period. All participation was voluntary of course, and each participant gave their informed consent. Owing to our longstanding positive engagement with the Hadza community, and owing to the minimally invasive nature of our research, it was possible for us to recruit subjects for this study in 15 camps, and no adverse events occurred during data collection.
Ethics oversight	Approval for this research was provided by all governing organizations (Institutional Review Boards at Harvard University, Yale University, Hunter College, the University of Arizona, University of California, Los Angeles; The Tanzania Commission for Science and Technology and the National Institute for Medical Research in Tanzania). All research participants provided their informed consent prior to participating in this project.

Note that full information on the approval of the study protocol must also be provided in the manuscript.
